# Combination of polythyleneimine regulating autophagy prodrug and Mdr1 siRNA for tumor multidrug resistance

**DOI:** 10.1186/s12951-022-01689-y

**Published:** 2022-11-11

**Authors:** Changduo Wang, Zhipeng Li, Ping Xu, Lisa Xu, Shangcong Han, Yong Sun

**Affiliations:** 1 Department of Pharmaceutics, School of Pharmacy, Qingdao University, Qingdao, 266071 China; 2 Department of Thoracic Surgery, the Second Affiliated Hospital of Qingdao University, Qingdao, 266000 China; 3 School of Public Health, Qingdao University, Qingdao, 266071 China

**Keywords:** MDR, Autophagy, siRNA, Tumor therapy, Prodrug

## Abstract

Multidrug resistance (MDR) has been restricting the efficacy of chemotherapy, which mainly include pump resistance and non-pump resistance. In order to fight overall MDR, a novel targeted gene/drug co-deliver nano system is developed, which can suppress the drug efflux pumps and modulate autophagy to overcoming both pump and non-pump resistance. Here, small interfere RNA (siRNA) is incorporated into polymer-drug conjugates (PEI-PTX, PP) which are composed of polyethyleneimine (PEI) and paclitaxel (PTX) via covalent bonds, and hyaluronic acid (HA) is coated on the surface of PP/siRNA to achieve long blood cycle and CD44-targeted delivery. The RNA interference to mdr1 gene is combined with autophagy inhibition by PP, which efficiently facilitate apoptosis of Taxol-resistant lung cancer cells (A549/T). Further study indicates that PEI in PP may play a significant role to block the autophagosome–lysosome fusion process by means of alkalizing lysosomes. Both in vitro and in vivo studies confirm that the nanoassemblies can successfully deliver PTX and siRNA into tumor cells and significantly inhibited A549/T tumor growth. In summary, the polymeric nanoassemblies provide a potential strategy for combating both pump and non-pump resistance via the synergism of RNAi and autophagy modulation.

**Supplementary Information:**

The online version contains supplementary material available at 10.1186/s12951-022-01689-y.

## Introduction

Multidrug resistance (MDR) is well recognized as one of the major obstacles that deteriorate the clinic effect of chemotherapy for non-small cell lung cancer (NSCLC) [1]. The development of MDR is complex, which can be generally divided into two major types, “pump” resistance and “non-pump” resistance [2]. For the pump resistance, it is raised by the overexpression of drug-efflux pumps, known as ATP-binding cassette (ABC) transporters, on the cell member to reducing intercellular drug concentration [3]. Among different types of ABC transporters, P-glycoprotein (P-gp), coded by the mdr1 gene, has been reported to overexpress in many cancer cells to induce MDR [4–6]. RNA interference (RNAi) technology is frequently combined with chemotherapy to suppress the expression of P-gp [7–9]. It takes advantage of small interfering RNA (siRNA) molecules to silence specific gene and regulate gene expression to obtain high specificity and excellent treatment effect [10, 11]. Polyethyleneimine (PEI), a cationic polymer, has strong nucleic acids compaction capacity due to the high density of amines. The “proton-sponge” effect of PEI would rupture endosomal and help gene translocate without degradation [12, 13]. Both branched PEI (*b*-PEI) and linear PEI (*L*-PEI) have been widely applied in RNAi. The *b*-PEI can result in greater internalization than *L*-PEI but also induce more cytotoxicity [14]. Meanwhile, high molecular weight PEIs have higher transfection efficiency and more side effects than low molecular weight (LMW) PEI [15, 16]. Thus, LMW *b*-PEIs (such as 1.8 k *b*-PEI or 10 K *b*-PEI) have been applied in gene delivery, which were modified to improve transfection, reduce the toxicity and functionalization (such as tumor-targeted, long blood circle, and drug loading) [17]. However, the down-regulation of P-gp via siRNA wouldn’t reverse non-pump resistance, which may limit the further improvement of chemotherapy.

Apart from pump resistance, non-pump resistance also contributes significantly to MDR [18]. For instance, autophagy as a pro-survival factor has a role in the development of MDR [19]. Autophagy is a lysosome-based degradative pathway activated in limited growth conditions, which could degrade cytoplasmic materials (damaged organelles, obsolete proteins, and invading pathogens) and recycle energy to maintain homeostasis in cells [20]. It is a double-edged sword for MDR tumors. Excessive autophagy could promote apoptosis and autophagic death of tumor cell which is also known as the type II programmed cell death [21]. What’s more, there are increasing evidences suggesting that autophagy protect cells under therapeutic stress and promote the development of MDR [22, 23]. Chloroquine (CQ) and hydroxychloroquine (HCQ), clinical antimalarial drugs, can block the fusion of autophagosomes with lysosomes via alkalizing lysosomes [24]. There are many studies co-delivering CQ (or HCQ) and chemotherapeutic drugs to sensitize the cancer cells [25–28]. However, the long-term use of CQ or HCQ are associated with various side effects, and the irreversible retinopathy caused by CQ (or HCQ) could remain develop after drug withdrawal [29–31]. Recently, nanoparticle-based autophagy inhibitors have attracted attentions in tumor MDR [32]. Gold nanoparticles are proven to block autophagic flux by impairing lysosome and induce autophagosome accumulation [33]. The pH-sensitive nanoparticles based on poly (β-amino ester) copolymers can lead to block autophagic flux and autophagic cell death under high concentrations [34]. The pH-sensitive polymer, mPEG-b-p(DPA-bDMAEMA), could self-assemble into micelles and be capable of loading chemotherapeutic agent, which unfold autophagic inhibition ability and high antitumor efficiency [35]. The autophagy inhibition facilitated by nanoparticles have also been successfully applied in reversing MDR, but there are few reports about suppressing both P-gp and autophagy to work on “pump” and “non-pump” resistance and to combat tumor MDR [2, 36, 37].

Herein, in order to reverse overall MDR, we designed a novel hyaluronic acid (HA)-coated siRNA/Paclitaxel (PTX) co-delivery nanoassemblies which can efficiently deliver paclitaxel and mdr1 siRNA, block autophagic flux and suppress P-gp level. We synthesized the polymer-drug conjugates of low molecular weight polyethyleneimine (1.8 K *b*-PEI) and PTX, named PEI-PTX (PP), which has high drug loading content (~ 26.2 wt%) and have ability to deliver gene. Subsequently, mdr1-siRNA was condensed within PP to form PP/ siRNA driven by electrostatic interaction. Finally, HA was coated on the surface of PP/siRNA to from stable nanoassemblies (PP/siRNA/HA). HA, viscous mucopolysaccharide, is widely used in nano drug delivery because of excellent biocompatibility and biodegradability, meanwhile it also can reduce the clearance of mononuclear macrophages and target CD44-overexpression tumor [38, 39]. With entering cells mediated by CD44-receptors, PP/siRNA/HA will be shattered within enrich-enzyme endo/lysosome. After the HA shell of nanoassemblies being degraded by HAase in endo/lysosome, exposed PP could rupture endo/lysosomal membrane and block autophagic flux to reverse non-pump resistance. Mdr1 siRNA (as well as PP) would be released from endo/lysosome via the “proton-sponge” effect where PTX would be cleaved from PP by nonspecific esterase in cytoplasm. The gene silencing by siRNA and autophagy modulation complemented each other, which improved the anti-cancer effect of PTX in Taxol-resistant non-small cell lung cancer cells (A549/T cells). We further demonstrate the PEI conjugate plays a significant role in autophagy modulation, which can alkalize and impair lysosomes to block autophagosome–lysosome fusion and lead to the accumulation of autophagosome. The potency of polymeric nanoassemblies is evaluated by A549/T cells and by A549/T tumor-bearing mice. Our work advances a novel strategy for MDR that could block autophagic flux, and achieve overcoming pump and non-pump resistance when combined with RNAi and chemotherapy.

## Experimental section

### Materials

Succinic anhydride, *b*-PEI (MW 1,800), *b*-PEI (MW 250,000), 2-(7-Azabenzotriazol-1-yl)-*N, N, N', N'*-tetramethyluronium hexafluorophosphate (HATU), N-ethyldiisopropylamine (DPIEA) et.al was purchased from Adamas-beta (Shanghai, China). All of solvent were purchased from Macklin (Shanghai, China). Chloroquine Phosphate was obtained from Sigma-Aldrich (St. Louis, MO, USA). Antibodies used for Western Blotting and immunofluorescence including rabbit anti-LC3B, anti-p62, anti-Pgp, goat anti-rabbit IgG (H + L) (HRP, Cora Lite 594) were obtained from Proteintech (Wuhan, China). Alexa fluor 647-labeled goat anti-rabbit IgG (H + L), rabbit anti-CD44, anti-ki67, Ad-GFP-LC3B, Lyso-Tracker Red, TUNEL Apoptosis Assay Kit, acid phosphatase assay kit et.al were purchased from Beyotime (Shanghai, China). All other reagents for western blotting and gel electrophoresis were obtained from Solarbio (Beijing, China).

Targeting human P-gp siRNA sequences:

Sense: 5’-AAGAAGGAAAAGAAACCAACUdTdT-3’;

Anti-sense: 5’-AGUUGGUUUCUUUUCCUUCUUdTdT-3’.

All of siRNA were obtained by GenePharma Co. Ltd. (Shanghai, China).

### Preparation of PEI-PTX (PP)

**Fig. 1:**
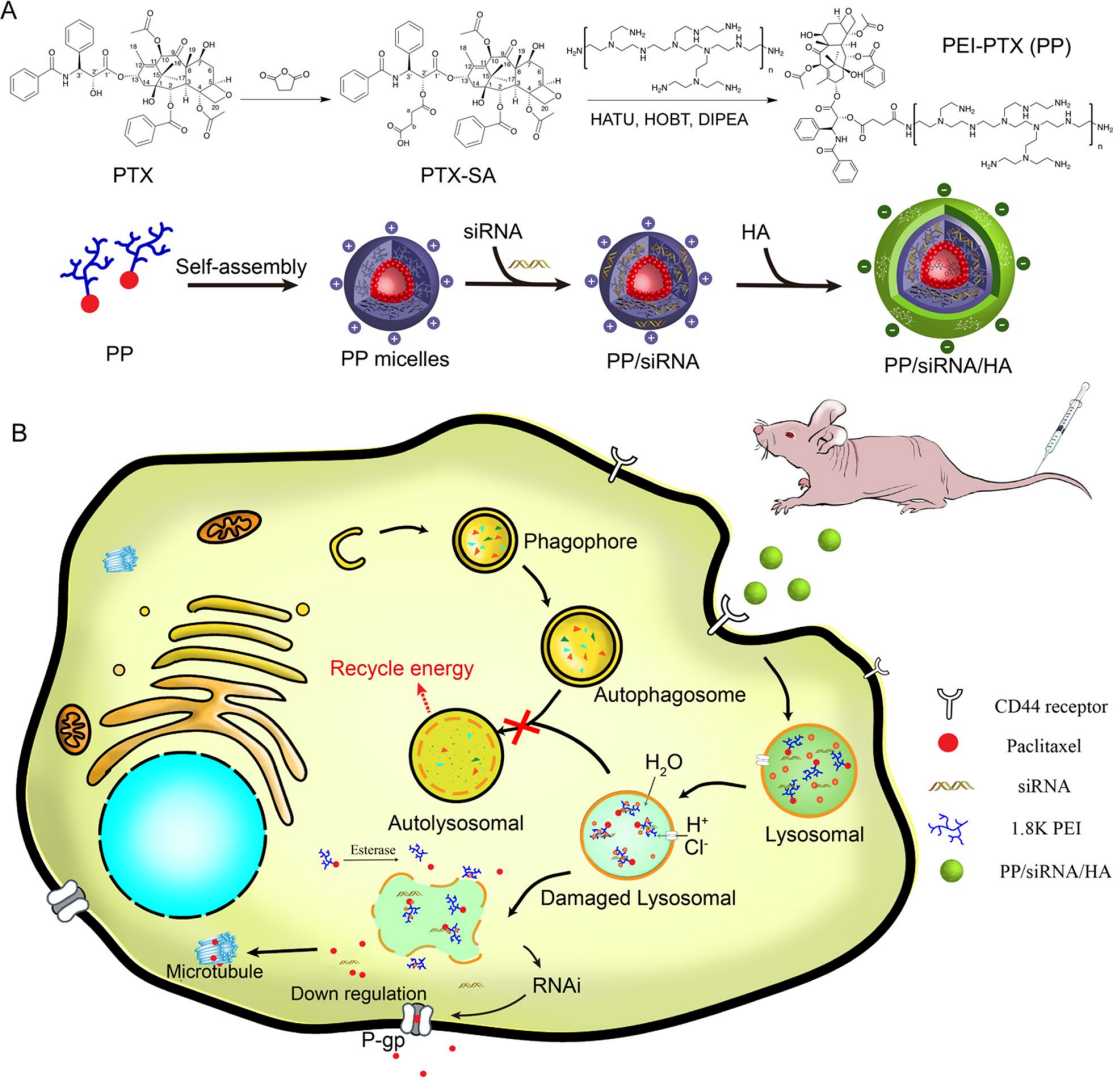
Schematic illustration displayed the strategy of siRNA/PTX co-delivery and the autophagic effects of nanoassemblies

Synthesis of PEI-PTX was carried in two steps, as shown in Fig. 1A. In the first step, PTX (100 mg/0.117 mmol) and succinyloxide (146 mg/1.459 mmol) were added in anhydrous pyridine for stirring at room temperature for 12 h. Then, the solvent was evaporated under reduced pressure, and deionized water was added for another 2 h. The pH was adjusted at 2–3 with HCl, and solution was extracted with ethyl acetate. The ethyl acetate extractant was washed with 0.2 M NaHCO_3_ and saturated salt water, respectively. The organic layer was dried over MgSO_4_. Removal of the solvents provided a white solid of PTX-SA (99.34 mg, productivity 89.1%). The formed PTX-SA was characterized by FT-IR, ^1^H NMR and mass spectrum.

In the second step, PEI-PTX was synthesized as follows: briefly, 200.0 mg PEI (MW 1800) was dissolved 5 mL water, and the pH was adjusted at 8 with HCL. The solution was lyophilized and dissolved in 5 mL DMSO for further use. PTX-SA (100 mg/ 0.11 mmol), HATU (76 mg/0.20 mmol), HOBt (28 mg/0.20 mmol) were dissolved in 1 mL DMSO, and DIPEA (130 mg/1.0 mmol) was added to the solution. The mixture was reacted at room temperature for 3 h. Then, the activated PTX-SA reaction solution was mixed with the PEI solution as mentioned above. After 24 h reaction, PEI-PTX solution was purified through dialysis (MWCO 3,500 DA) and ethyl acetate washing, and was lyophilized to obtain a bright yellow spongy solid. The product was characterized by FT-IR, ^1^H NMR, and gel permeation chromatography (GPC). GPC was performed on Agilent Technologies 1260 Infinity equipped with refractive index detector, with DMF as the eluent at a flow rate of 1.0 mL/min at 35℃; the column was TOSOH TSKgel G2000HHR.

### Preparation and characterization of nanocomplex

**Fig. 2:**
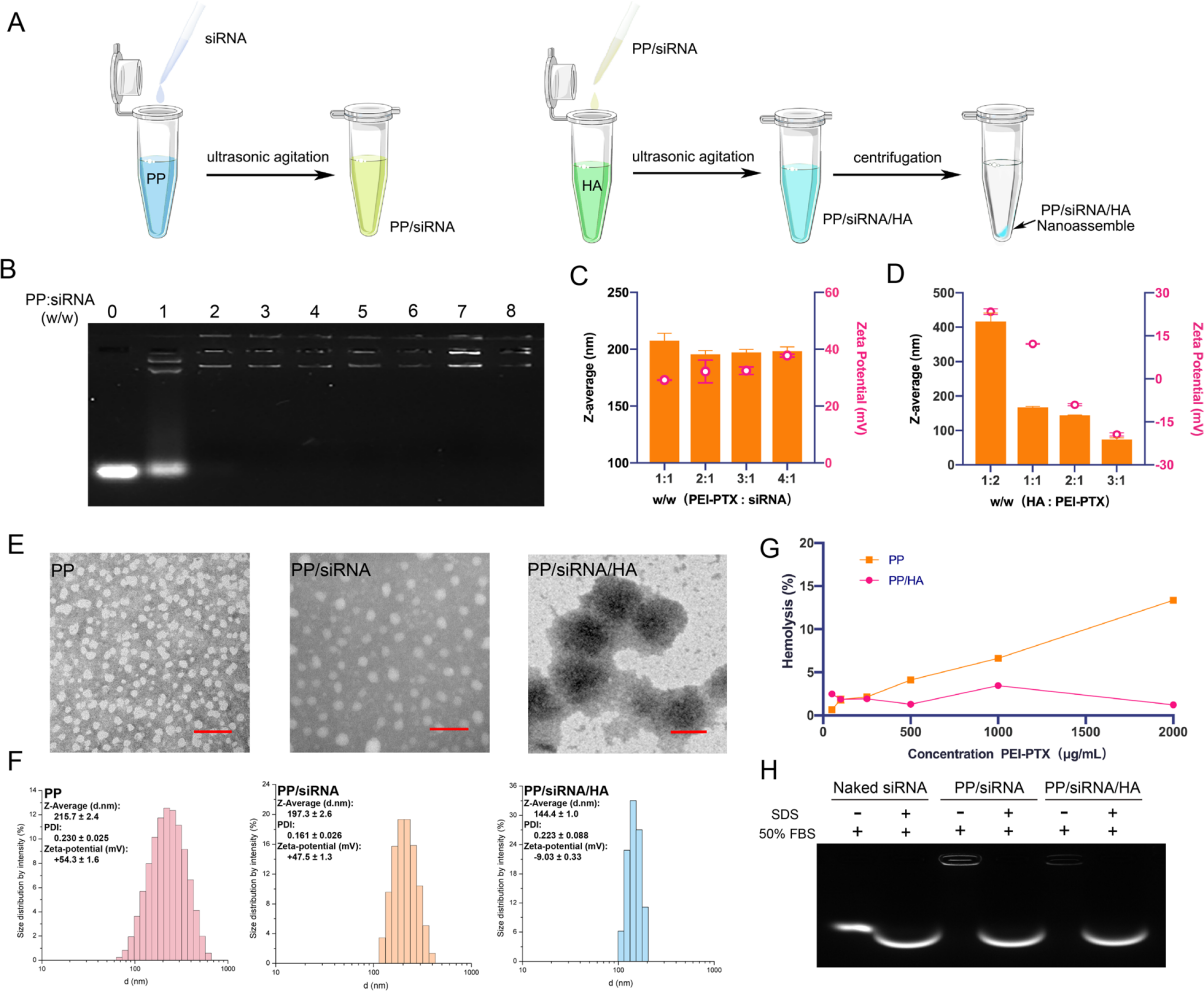
Preparation, characterization, and stabilities of the nanoassemblies. **A** Schematic of PP/siRNA/HA nanoassemblies. PP: PEI-PTX conjugates; PP/siRNA: PP with siRNA condensed on the surface; PP/siRNA/HA; PP/siRNA covered with HA. **B** Gel electrophoresis of PP/siRNA at various weigh ratios (**C**) The ζ-potential and z-average of PP/siRNA at different ratio of PP and siRNA (**D**) The ζ-potential and z-average of PP/siRNA/HA at different ratio of PP/siRNA and HA. **E** TEM images of PP, PP/siRNA, and PP/siRNA/HA; visualized by negative staining with 1% phosphotungstic acid. Scale bars: 100 nm. **F** Size distribution of PP, PP/siRNA, and PP/siRNA/HA. **G** Hemolytic toxicity study of PP and PP/HA. **H** The stabilities of PP/siRNA and PP/siRNA/HA after incubated in 50% FBS. The data are presented as means ± SD

As illustrated in Fig. 2A, 1 mg PP was dissolved in was dissolved in 1 mL deionized water and mixed with siRNA at a proper ratio under ultrasonic agitation (40 kHz, 25 ℃). The 1 mL siRNA-loaded PP (PP/siRNA, 1 mg/mL) was coated with HA by incubation in 10 mL HA solution under ultrasonic agitation (40 kHz, 25 ℃). Finally, the HA-coated, siRNA-loaded PP (PP/siRNA/HA) were collected by centrifugation (12,000 r, 20 min). PP/siRNA and PP/HA were also prepared and collected with above methods.

After diluting the micelle solution with distilled water, the mean particle diameter (Z-average) and zeta potentials of the nanomicelles were determined via Malvern Zetasizer Nano ZS90 (British Malvern Instrument Co. Ltd). The morphological features of PP, PP/siRNA and PP/siRNA/HA were observed by using a transmission electron microscope (TEM, JSM-6490LA, Japanese company JEOL). Briley, a copper grid was immersed in a pre-diluted micellar solution for 3–5 min and then stained with 1% phosphotungstic acid after air-drying. The TEM images of the samples were taken after being dried again with an incandescent lamp.

### Hemolytic toxicity study

To assess the potential hemolytic toxicity of PP and PP/HA, a red blood cell suspension was diluted to 2% with phosphate buffer solution (pH7.4). Different concentrations of PP and PP/HA were dispersed in the 2% RBC suspension (1:1, volume ratio). In addition, TritonX-100 and physiological saline were used as the positive control and negative control, respectively. All samples were incubated in a constant temperature water bath at 37 ℃ for 3 h and then centrifuged at 4000 rpm for 15 min. The supernatant was collected, and its absorbance was measured at 540 nm using a microplate reader. The percentage of hemolysis was calculated by taking the absorbance of the TritonX-100 sample as the hemolysis rate of 100%. The hemolysis rate was calculated according to the following equations:

$${\text{Hemolysis}}\,{\text{rate}}\,\left( \% \right) = \,\frac{{{\text{A}}_{{{\text{Nanoparticles}}}} - {\text{A}}_{{{\text{saline}}}} }}{{{\text{A}}_{{{\text{TritonX}} - 100}} - {\text{A}}_{{{\text{saline}}}} }} \times 100\%$$

### The drug loading and in vitro PTX release

The PTX loading of PP, PP/siRNA, PP/HA, PP/siRNA/HA were determined by high-performance liquid chromatography (HPLC, 1260 Infinity, Agilent) after the hydrolysis in methanol/water (1:1) solution at 37 ℃ for 24 h. The accumulative release of PTX from PP was conducted in the release medium (containing 1% Tween-80) of pH5.0 or 7.4 PBS, with or without 0.4 IU/mL esterase at 37 ℃, under a shaking at the speed of 100 rpm/min. The accumulative release of PTX from PP/siRNA/HA NPs was conducted in the release medium (containing 1% Tween-80) of pH7.4 or 6.0 PBS, with or without 0.4 mg/mL HAase at 37 ℃, under a shaking at the speed of 100 rpm/min. The dialysis bag (MW = 1000 Da) containing 0.5 mL solution of PP (1 mg/mL) or PP/siRNA/HA NPs (3.6 mg/mL) was put into 10 mL release media. 0.2 mL release media was withdrawn and determined at the selected time. The HPLC was applied to determine the content of PTX (mobile phase: acetonitrile/water = 50/50). During the whole process, the UV detection was set at 227 nm, the flow rate was 1 mL/min and the temperature of C18 column was 30 ℃.

### Cytotoxicity assay

Human lung cancer cell lines, A549 and Taxol-resistant A549 (A549/T) were purchased from Procell Life Science&Technology Co.,Ltd (Wuhan, China). All cells were cultured in F-12 K media containing 10% FBS and 100 IU/ml penicillin, 100 mg/ml streptomycin and 2 mM L-glutamine. All cells were maintained in a 37 ℃ incubator with 5% CO_2_ for further treatment. The cell viability/cytotoxic potential of individual formulation were performed by MTT assay. Briefly, cells were seeded into at a seeding density of 5000 cells/96-well plate and incubated for 24 h. Following day, medium was removed and cells were incubated with free PTX, PP, PP/HA, PP/siRNA, and PP/siRNA/HA at different concentrations of PTX (ranged from 10^–5^ to 1 μg/mL) and incubated for 48 h. At designated time intervals, cells were treated with 100 μL/well MTT solution (0.5 mg/ml in serum free media) and incubated for 4 h. The purple blue formazan crystals were extracted by the addition of DMSO (150 μL/well) and absorbance was measured by microplate reader (Sunrise, TECAN). The IC_50_ value of PTX in these cells was calculated by GraphPad Prsim software.

### Cell uptake study

Cellular uptake by flow cytometer analysis The A549 cells, as well as A549/T cells, were seeded at a density of 2 × 10^5^ cells/6-well plate and allowed to attach for 24 h. The cells were exposed to PP/FAM-siRNA/HA (containing 40 nM FAM-siRNA) and incubated for 1 and 4 h. The cells were washed twice with PBS, trypsinized, collected and resuspended in PBS. The amount of cellular uptake was confirmed by flow cytometry (Beckman coulter life sciences, CytoFLEX, USA).

The cellular uptake of drugs was observed by confocal laser scanning microscope (CLSM). In order to make the PP/siRNA/HA NPs have fluorescence signal, we attempted to label FAM-siRNA to NPs. The cell nuclei were stained by DAPI. During the whole process, in short, 2 × 10^4^ A549 cells, as well as A549/T cells, were seeded into 24-well plates and incubated for 24 h. The media was removed and the cells were cultured with FAM-siRNA (40 nM) loaded in PP/siRNA/HA NPs. After 1 h and 4 h, the cells were washed, fixed, stained, and ultimately observed by CLSM.

### Apoptosis assay and cell-cycle analysis

The A549 and A549/T were seeded at a density of 3 × 10^5^ cells/6-well plate and allowed to attach for 24 h. The cells were treated with free PTX, PP, PP/HA, PP/siRNA and PP/siRNA/HA (containing 10 ng/mL PTX), and then incubated for 24 h at 37 ℃ in a standard incubator. A control was maintained as untreated cells. After the incubation period, cells were washed, trypsinized, collected and resuspended in a 195 μL of binding buffer. Immediately, 5 μL of annexin V-FITC and 10 μL of propidium iodide (PI) was added and gently vortexed and kept aside for 30 min. The proportions of apoptotic or stained cells were observed by flow cytometer.

3 × 10^5^ cells A549 and A549/T cells were seeded into 6-well plate and cultured overnight respectively. Then, the media was replaced and cells were cultured with free PTX, PP, PP/HA, PP/siRNA and PP/siRNA/HA (containing 10 ng/mL PTX). The cells were collected, fixed (70% ethanol V/V, − 20 ℃, overnight) and stained with PI (50 μg/mL, 20 min, 4 ℃) for the further analysis of cell cycle distribution by flow cytometry.

### In vitro siRNA transfection and analysis of P-gp expression

The A549 and A549/T cells were seeded at a density of 3 × 10^5^ cells/6-well plate. After reaching 70% confluence, the cells were incubated with control (fresh media), PP, PP/HA, PP/siRNA, and PP/siRNA/HA (containing 100 nM siRNA) for 48 h (mRNA extraction) or 72 h (protein isolation). The intracellular mRNA level and protein content were detected by reverse transcription PCR (RT-PCR) and Western blot, respectively.

Total RNA from cells was extracted using the Total RNA Extraction Kit (Solarbio, China) according to the manufacturer's protocol. The concentration of extracted RNA was determined with NanoDrop One (Thermo scientific, USA). The cDNA was produced using the MonScript™ RTIII All-in-One Mix with dsDNase (Monad Biotech, China). The reverse-transcribed cDNA was used for PCR amplification using MonAmp™ ChemoHS qPCR Mix (Monad Biotech, China).

The primer sequences for MDR1-mRNA amplification:

Forward 5′-AGGAAGCCAATGCCTATGACTTTA-3’;

Reverse 5′-CAACTGGGCCCCTCTCTCTC-3’.

The primer sequences for GAPDH amplification:

Forward 5′- AAATCAAGTGGGGCGATGCTG -3’;

Reverse 5′- GCAGGAGGCATTGCTGATGAT -3’.

GAPDH was used as a reference gene. PCR was performed on ABI StepOne Plus™ (Applied Biosystems^®^, USA).

For western blotting, the cells were lysed using RIPA buffer (Solarbio, China) after treatment. The protein content was determined by BCA Protein Assay Kit (Solarbio, China). For all western blots, samples containing 50–150 μg of total protein were separated by SDS-PAGE on 10–15% gel. The separated polypeptides were transferred to polyvinylidene fluoride (PVDF) membrane, probed with antibodies, and visualized by ECL with ChemiDoc™ XRS + (Bio-Rad Laboratories, USA) as previously described. All primary antibodies were purchased from Proteintech^®^ (Wuhan, China) with 1:500 dilutions.

### In vitro autophagy modulation study

The A549/T cells were seeded on 6-well plate. The cells were treated with PP (different concentrations ranged from 0.1 to 50 μM), negative control (PBS), and positive control (10 μM CQ), respectively. The intracellular LC3/p62 proteins were detected by Western blotting.

For immunofluorescence, after treatment, the A549/T cells expressing GFP-LC3B were fixed using 4% paraformaldehyde, washed in PBS, permeabilized with 0.1% Triton X-100, and blocked in 5% goat serum. Cells were then stained with rabbit anti-p62 antibody and secondary antibody (Alexa Fluor^®^ 647 Conjugate) followed by DAPI nuclear stains. For co-localization analysis, the cells expressing GFP-LC3B were fixed and stained nuclear after treatment of Cy5-siRNA loaded nanoassemblies (PP/Cy5-siRNA/HA). All stained samples were visualized under CLSM (Leica, USA).

For observation of autophagosome, A549/T cells were collected using cell scraper and centrifuged after treatment, and the cell sediments were fixed in 2.5% glutaraldehyde for at least 24 h. Then, the cells were graded dehydrated and fixed in epoxy. Ultrathin sections of the cells were examined by transmission electron microscope.

### Gel retardation assay for testing siRNA-loading capacity and siRNA stability

The siRNA-loading capacity of PP was evaluated by the agarose gel retardation assay. The siRNA-loaded PP (PP/siRNA) complexes (containing 0.75 μM siRNA) were prepared by varying the PP/siRNA weight ratio from 1/1 to 8/1. The complexes were loaded in 1% agarose gel and run in 1 × TAE buffer at 120 V for 15 min. The gel was stained with SuperRed/GelRed (Biosharp, China), and siRNA bands were visualized with ChemiDoc™ XRS + (Bio-Rad Laboratories, USA).

For serum stability testing, siRNA or NPs (containing 0.75 μM siRNA) were challenged with 50% FBS at 37 °C for different time (0 to 24 h) and analyzed by agarose gel electrophoresis. Heparin, as an ion exchange ligand, is widely applied in heparin-affinity column to pure various biomacromolecules (such as proteins, nucleic acid, and lipoprotein) [40]. Heparin can displace siRNA from cationic gene carriers via ion exchange. For the anion resistance of PP/siRNA and PP/siRNA/HA (containing 0.75 μM siRNA), various dosage of heparin (heparin/siRNA range 1 to 8 IU/μg) were used to replace siRNA from complexes at 37 °C for 2 h.

### In vivo biodistribution

BALB/c nude mice bearing A549/T tumor were used to study the biodistribution of PP/siRNA/HA nanoassemblies via fluorescence imaging [41]. When the tumor volume was about 300 mm^3^, Cy5-siRNA solution or PP/Cy5-siRNA/HA nanoassemblies were intravenously administrated (1 mg/kg equal to Cy5-siRNA). The mice were observed with in vivo fluorescence imaging system at 1, 3, 6, 12 and 24 h after injection (n = 3). Furthermore, at 24 h after injection, the mice were dissected and the heart, liver, spleen, lungs, kidneys, and tumors were used for imaging.

### In vivo antitumor efficacy

Balb/c nude mice (male, 3 − 4 weeks) were purchased from Beijing Vital River Laboratory Animal Technology Co. Ltd., China. After one week of adaptation, tumor bearing mice were established by subcutaneous injection of 1 × 10^7^ A549/T cells suspended in in PBS and Matrigel media mixed at 1:1 ratio. When the tumor size reached 100 mm3, mice were randomly classified into 4 groups (n = 6): untreated control (0.9% NaCl), free PTX (Taxol), PP/HA, and PP/siRNA/HA. Then, these formulations were administrated via tail vein (5 mg/kg/time^−1^ of PTX, 5 mg/kg/time^−1^ of siRNA) for five injections every four day. Tumor size was monitored every 2 days. The length (L) and width (W) of each tumor were measured by a digital caliper, and the volume (V) was calculated by the modified ellipsoid formula: V = (L × W^2^)/2. At the 21th day of observation, all of mice were sacrificed and its tumors were weighed. The tumor burden was calculated as: Tumor burden (%) = (W_tumor_/W_mice_) × 100. Moreover, the heart, liver, spleen, lung, kidney, and tumor were dissected for H&E staining to evaluate physiological changes of main organs and tumors. TUNEL and Ki67 fluorescence staining were used to test the apoptosis and proliferation of tumor. The immunofluorescence staining of P-gp, LC3B, and p62 were valuated to the gene silencing and autophagy modulation of nanoassemblies in vivo.

### Statistical analysis

All data in the study are shown in means ± SD. The unpaired Student’s t test (two-tailed) was used for two-group comparison with *p < 0.05, **p < 0.01, and ***p < 0.001 as indicative of statistically significant differences. The GraphPad Prism software was used for data analysis and visualization.

## Results and discussion

### Synthesis and characterization of PEI-PTX (PP) polymers

The procedure for synthesizing PP polymers is shown in Additional file 1: Figure S1. PTX-SA was obtained by conjugating succinic acid to the 2’-OH group of PTX via ester bond formation. As showed in Additional file 1: Figure S2, the wide and strong peak of 3438.38 cm^−1^ in the spectra of PTX-SA was originated from the hydrogen bond association hydroxide, suggesting the existence of succinate. The ^1^H NMR spectrum and mass spectrum of PTX-SA was showed in Additional file 1: Figure S3B and S4. HRESI-MS: *m/z* 976.72 [M + K]^+^, C_51_H_55_NO_16_. ^1^H NMR (400 MHz, DMSO-d_6_), δ (ppm): 12.28 (1H, s, b-COOH), 9.22 (1H, d, J = 8.5 Hz, 3’-NH-), 7.99–7.19 (15H, Ar–H), 6.29 (1H, s, 10-H), 5.81 (1H, t, J = 8.9 Hz, 13-H), 5.54 (1H, t, J = 8.7 Hz, 3’-H), 5.34 (1H, d, J = 9.0 Hz, 2-H), 4.93 (1H, m, 5-H), 4.64 (1H, s, 2’-H), 4.12 (1H, q, J = 6.8 Hz, 7-H), 4.02 (2H, q, J = 8.3 Hz, 20-H), 3.57 (1H, d, J = 7.1 Hz, 6-αH), 2.62 (2H, t, J = 6.4 Hz, a-H), 2.32 (2H, m, b-H), 2.24 (3H, s, 4-COCH_3_), 2.11 (3H, s, 10-COCH_3_), 1.87–1.79 (2H, m, 14-H), 1.76 (3H, s, 18-H), 1.63(1H, m, 6-βH), 1.50 (3H, s, 19-H), 1.02 (3H, s, 17-H), 1.00 (3H, s, 16-H). Meanwhile, compared with the ^1^H NMR spectrum of PTX (Additional file 1: Figure S3A), the peak assigned to be the hydroxyl protons contributing to the 2’-OH groups at δ 6.16 disappeared, which demonstrates the full derivatization of all the 2’-OH groups.

Then, PP was synthesized via amide reaction. The intensity of infrared absorption peak, generally, is positive related with the change of dipole moment. In the spectrum of PEI-PTX (Additional file 1: Figure S5), the peak at 711.12 cm^−1^ was assigned to arene’s γ_CH_ of PTX, the peak at 1245.07 cm^−1^ was assigned to oxhydryl’s υ_CO_ of PTX, and the peak at 1112.84 cm^−1^ and 1069.34 cm^−1^ were assigned to primary (and secondary) amine’s υ_CN_ of PEI. The peaks at 1580.97 cm^−1^ and 1461.58 cm^−1^ in the spectrum of PEI were assigned to primary (and secondary) amine’s δ_NH_ of PEI. However, the peaks at 1580.97 cm^−1^ and 1461.58 cm^−1^ in the spectrum of PEI were not shown in the spectrum of PEI-PTX. The primary amine of PEI were shift to amide, leading the peak at 1580.97 cm^−1^ being blue shifted and integrated to the peaks at 1648.84 cm^−1^. In addition, because the secondary amine hydrogens of PEI were formed hydrogen bonds during the synthesis process, the peak at 1461.58 cm^−1^ was red shifted and integrated to the peaks at 1245.07 cm^−1^. In the spectra of PTX-SA, the absorption peak at 1722.69 cm^−1^ were assigned to amide’s and ester’s υ_C=O_, and the absorption peak at 1641.02 cm^−1^ was assigned to amide’s δ_NH_. In addition, The N–H bending vibration (δ_NH_) is an important characteristic of amine. After the amide reaction between carboxyl of PTX-SA and primary amine of PEI, the intensity of amide’s δ_NH_ peak at 1648.84 cm^−1^ was increased because of the generation of new amide. Thus, the FT-IR results show the amide reaction between PTX-SA and PEI was successfully synthesized. Besides, according to ^1^H NMR (400 MHz, DMSO-d_6_) (Additional file 1: Figure S6), the peaks of δ 2.40 and δ 2.54 were assigned to PEI, and the peaks of PTX in PP were assigned as following: 9.22 (1H, d, J = 9.0 Hz, 3’-NH-), 8.05–7.10 (15H, Ar–H), 6.30 (1H, s, 10-H), 5.90 (1H, t, J = 5.9 Hz, 13-H), 5.56 (1H, m, 3’-H), 5.41 (1H, m, 2-H), 4.92 (1H, d, J = 4.9 Hz, 5-H), 4.62 (1H, m, 2’-H), 4.11 (1H, m, 6-αH), 4.01 (2H, dd, 20-H), 2.23 (s, 4-COCH_3_), 2.12 (s, 10-COCH_3_), 1.80 (1H, m, 14-H), 1.71 (3H, s, 18-H), 1.65(1H, m, 6-βH), 1.51 (3H, s, 19-H), 1.02 (6H, s, 16,17-H). The δ 9.05 in the ^1^H NMR spectrum of PP was originated from 3’-NH- in PTX, which was lower than of PTX-SA (δ 9.22) because PEI weakened the deshielading effect of succinate. Meanwhile, there is no peak assigned to 2’-OH at δ 6.16, which supported the conjugation between PTX and PEI via 2’-ester bond. The PTX content of PP was determined according to the ^1^H NMR spectra by comparing integrals of peaks at δ 8.05–7.15 (aromatic protons of PTX) with δ 2.45–2.35 (partial methylene protons of PEI), which indicated that PP had 27.43 wt% PTX (calculation method showed in Additional file 1: Figure S6). GPC measurement showed that PTX was covalently bonded with PEI to form polymer-drug conjugates (Additional file 1: Figure S7). However, there are the bimodal distribution and of PP indicated that one or more PTX would be grafted into PEI, and the cross-linking of PP may have caused the distribution at low elution volumes.

### Preparation and characterization of PP/siRNA/HA nanoassemblies

The process of self-assembly was shown in Fig. 2A. Initially, the PP copolymer dissolved in Milli-Q water can be self-assembled into micelles under sonication. Dynamic light scattering (DLS) analysis showed that the hydrodynamic diameter of PP micelles was 215.7 ± 2.4 nm (Fig. 2F), and the transmission electron microscopy (TEM) images showed that the micelles were spherical (Fig. 2E). As shown in Additional file 1: Figure S7, the critical micelle concentration (CMC) of PP micelles was 6.3 × 10^–3^ mg/mL, indicating the excellent thermodynamic stability of micelles. To determine the optimal weight ratio (PP/siRNA) for siRNA delivery, we used agarose gel electrophoresis to evaluate the siRNA encapsulation of PP (Fig. 2B). It was found that binding capacity increased with increasing the ratio of PP, and all of siRNA were encapsulated when the weight ratio of PP: siRNA increased to 3:1. The encapsulation efficiency of siRNA was up to 92.68 ± 0.78% at 3:1 weight ratio and maintained unchanged when further increasing ratios (Additional file 1: Figure S9). As shown in Fig. 2C, an increasing tendency of zeta potential was detected during the increase of weight ratios, further indicating the successful loading of siRNA. Therefore, the optimal weight ratio (PP/siRNA, w/w) of 3:1 was used for further anti-tumor study considering the encapsulation efficiency, z-average size, and zeta potential. The siRNA-loaded PP (PP/siRNA) showed a reduction of positive charge (from + 54.3 ± 1.6 mV of PP to + 47.5 ± 1.3 mV of PP/siRNA) and the slight decrease of particle size (from 215.7 ± 2.4 nm.

to 197.3 ± 2.6 nm), indicating the condensing of siRNA on the PP surface (Fig. 2F). Subsequently, PP/siRNA was coated with a HA shell by electrostatic interaction. To determine the optimal ratio of HA and PP, we measured the z-average size and zeta potential of HA-coated PP/siRNA (PP/siRNA/HA) with different (HA: PP) ratios ranged from 1:2 to 3:1. The surface charge of PP/siRNA/HA emerged reversal from positive charge to negative when the weight ratio of HA: PP increased to 2:1, and the particle size was decreased during the increase of HA ratio (Fig. 2D). Considering longer blood circulation and higher tumor targeting, we used 2:1 as optimal weight ratio of HA/PP (w/w) for further study. PP/siRNA/HA showed a gelatinous shell on surface in TEM (Fig. 2E), a reversal of zeta potential (from + 47.5 ± 1.3 mV of PP/siRNA to − 9.0 ± 0.3 mV of PP/siRNA/HA), and the decrease of particle size (from 197.3 ± 2.6 nm of PP/siRNA to 144.4 ± 1.0 nm of PP/siRNA/HA) (Fig. 2F), revealing the presence of HA shell. The photos of nanoassemblies PBS solutions were showed in Additional file 1: Figure S10, there were translucent, azure, well-distributed colloidal solutions in PBS. To confirm the drug loading contents (DLs) of PTX, different preparations were hydrolyzed, and then the content of PTX was measured by HPLC. The PP exhibited high drug loading efficiency (25.15 ± 0.90%, wt%), and the DLs of PP/siRNA, PP/HA, and PP/siRNA/HA were 17.44 ± 0.43%, 7.27 ± 0.19% and 6.99 ± 0.15%, respectively (Table 1). Notably, the PTX content of PP determined by HPLC was slightly lower than by ^1^H NMR, which may be due to the incomplete release of PTX from PP micelles.

**Table 1:** Loading efficiency of PP, PP/siRNA, PP/HA, and PP/siRNA/HA (n = 3)

	PP	PP/siRNA	PP/HA	PP/siRNA/HA
Loading PTX Efficiency (wt %)	25.15 ± 0.90	17.44 ± 0.43	7.27 ± 0.19	6.99 ± 0.15

The PTX release was investigated in vitro simulated environment. As shown in Figure S13 (A), the succinic acid ester in PP could be hydrolyzed under acid condition or esterase catalysis. Compared with pH7.4 group, PP could release PTX at the presence of esterase, which indicated that PP would release PTX by nonspecific esterase in cytoplasm. PP/siRNA/HA nanoassemblies remained stable and no drug leakage under pH5.0 and pH7.4, but hyaluronidase (1%, w/w) could promote the drug release under acid condition, which indicated that hyaluronidase degraded HA shell. These results demonstrated that the succinic ester in PP can be hydrolyzed by nonspecific esterase and release of PTX in cytoplasm after PP escaping from endo/lysosomes to cytoplasm via the proton sponge mechanism.

### Stability in vitro

The stability of nanoparticle system is vital for its application in vivo [42]. Both PP/siRNA and PP/siRNA/HA (containing 0.75 μM siRNA) in 50% FBS showed no free siRNA band upon gel electrophoresis (Fig. 2H). This result demonstrated that the HA shell contributed to the stable encapsulation of siRNA. To test whether the HA shell protects siRNA from anionic environment in physiological fluids and enzymatic challenge in FBS, PP/siRNA and PP/siRNA/HA (containing 0.75 μM siRNA) were subjected to 50% FBS. PP and PP/siRNA/HA remained stable for 24 h and showed no signs of degradation due to the presence of serum nucleases, whereas naked siRNA was completely degraded in 12 h (Additional file 1: Figure S11A). Heparin can displace siRNA from cationic gene carriers via ion exchange [43]. As shown in Figure S11B, PP/siRNA/HA showed stronger heparin resistance ability than samples without HA-coated, further supporting the protective effect of the HA shell. Solubilizers, particularly cationic surfactants, usually cause severe hemolytic reaction when injected into blood vessel [44]. Thus, we conducted a hemolysis test to evaluate the safety of the PP and PP/HA. The PP cause severe hemolysis as we expected, but the PP/HA did not cause hemolysis at a high concentration and there was no significant difference when compared to the saline group (Fig. 2G and Additional file 1: Figure S12), which demonstrated the HA shell not only enhanced its stability, but also improved the safe and biocompatibility of nanoparticles. Thus, HA-coated PP could be used for intravenous injection. Considering the severe hemolysis and unsafety of PP without HA-coated, we used the PP/HA or PP/siRNA/HA as main preparations for further anti-tumor study in vivo.

### Cell uptake and endocytosis study

**Fig. 3:**
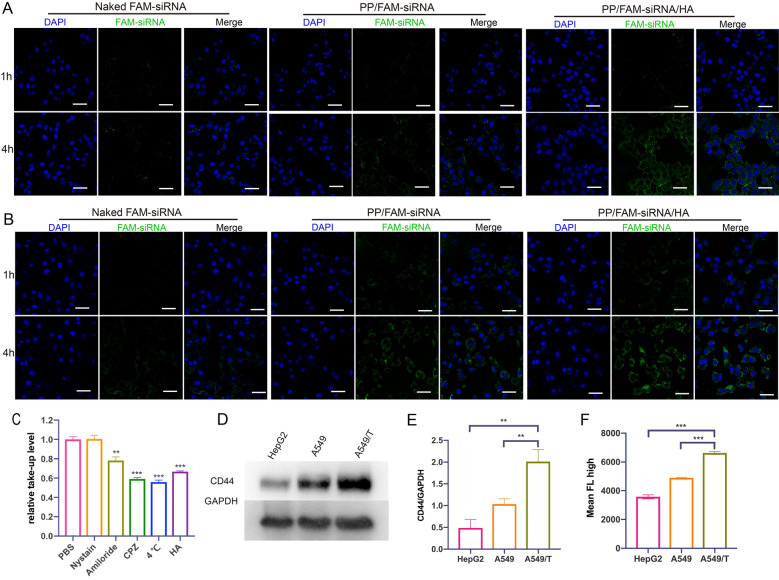
Internalization of nanoassemblies. CLSM images of A549 cells (**A**) and A549/T cells (**B**) incubated with naked FAM-siRNA, PP/FAM-siRNA or PP/FAM-siRNA/HA nanoassemblies for 1 h and 4 h. Cell nuclei were stained with DAPI. Scale bars: 50 μm (**C**) Endocytosis study of PP/siRNA/HA in A549/T cells by Flow cytometry (**D**) Western blot of P-gp expression in HepG2 cells, A549 cells, and A549/T cells. **E** Quantitive presentation of Western blotting. **F** Flow cytometry analysis of uptake in HepG2 cells, A549 cells, and A549/T cells after treated with PP/FAM-siRNA/HA for 4 h. The data are presented as means ± SD. **p < 0.01 and ***p < 0.001

Efficient cellular uptake is vital for gene transfection [41]. The confocal laser scanning microscope (CLSM) images of the FAM-siRNA (40 nM) in A549 and A549/T cells for 1 h and 4 h were shown in Fig. 3A and B. Naked FAM-siRNA, in either A549 cells or A549/T cells, exhibited weak fluorescence intensity at 1 h and 4 h since nucleic acid can hardly pass through cell membrane [45]. The green fluorescence was obviously brighter after incubated with PP/FAM-siRNA or PP/FAM-siRNA/HA for 4 h, indicating siRNA-loaded nanoassemblies could promote gene internalization. However, the fluorescence intensities at 4 h PP/FAM-siRNA/HA were stronger than PP/FAM-siRNA because PP/FAM-siRNA was unstable under rich serum environment. Meanwhile, as incubation time was prolonged, the green fluorescence signals became stronger, indicating that more nanoassemblies were internalized into cells. This phenomenon was also verified by quantitative evaluation by flow cytometry (Additional file 1: Figure S14). The results confirmed that the PP/siRNA/HA could promote the uptake of siRNA compared to naked siRNA. Endo/lysosomal escape capacity, avoiding the acid hydrolysis of gene, of FAM-siRNA loaded PP/siRNA/HA was evaluated by CLSM in either A549 or A549/T cells. As shown in Additional file 1: Figure S15, we could observe that the FAM-siRNA loaded nanoparticles were mainly entrapped in endo/lysosomal compartments at 1 h after uptake, while most FAM-siRNA could successfully escape into the cytoplasm at 4 h. The colocalization analyzes and its Pearson’s coefficient were further confirm this result. Therefore, the PP/siRNA/HA can exhibit excellent cellular internalization and endo/lysosomal escape ability, and could be used in gene and protein drug delivery.

The internalization of PP/siRNA/HA nanocomplexes was investigated by A549/T cells following treatment with caveolin-mediated endocytosis inhibitors (nystatin), clathrin-mediated endocytosis inhibitors (chlorpromazine), macropinocytosis inhibitors (amiloride), and energy inhibition (4 °C), respectively [46]. As displayed in Fig. 3C, the endocytosis of nanocomplexes was significantly inhibited under microthermal condition (4 °C), demonstrating the energy-dependent endocytosis mechanisms. The results in Fig. 3C revealed that nanoparticle uptake was significantly inhibited by chlorpromazine and amiloride treatment, suggesting that clathrin-mediated endocytosis and macropinocytosis may both play a role in the uptake of PP/siRNA/HA. Meanwhile, there is an additional group of overdose HA blocking CD44-receptor to investigate the uptake of PP/siRNA/HA, indicating that the endocytosis would be CD44-mediated endocytosis pathway. Hyaluronic acid, as reported, can enhance the internalization of nanocomplexes through binding CD44 receptor [47]. The CD44 expression of A549 cells, A549/T cells, and HepG2 cells (as CD44-negative cells) were detected by Western blotting (Fig. 3D and E). This result demonstrates that the CD44 expression of A549/T cells was significantly higher than A549 cells and HepG2 cells. To evaluate the CD44-mediated internalization, we investigated the uptake of PP/siRNA/HA by flow cytometry (Fig. 3F). The fluorescence intensity in A549/T cells was statistically higher than A549 and CD44-negative HepG2, suggesting the high potential targeting ability of HA-coated nanocomplexes.

### Silences gene expression in vitro

**Fig. 4:**
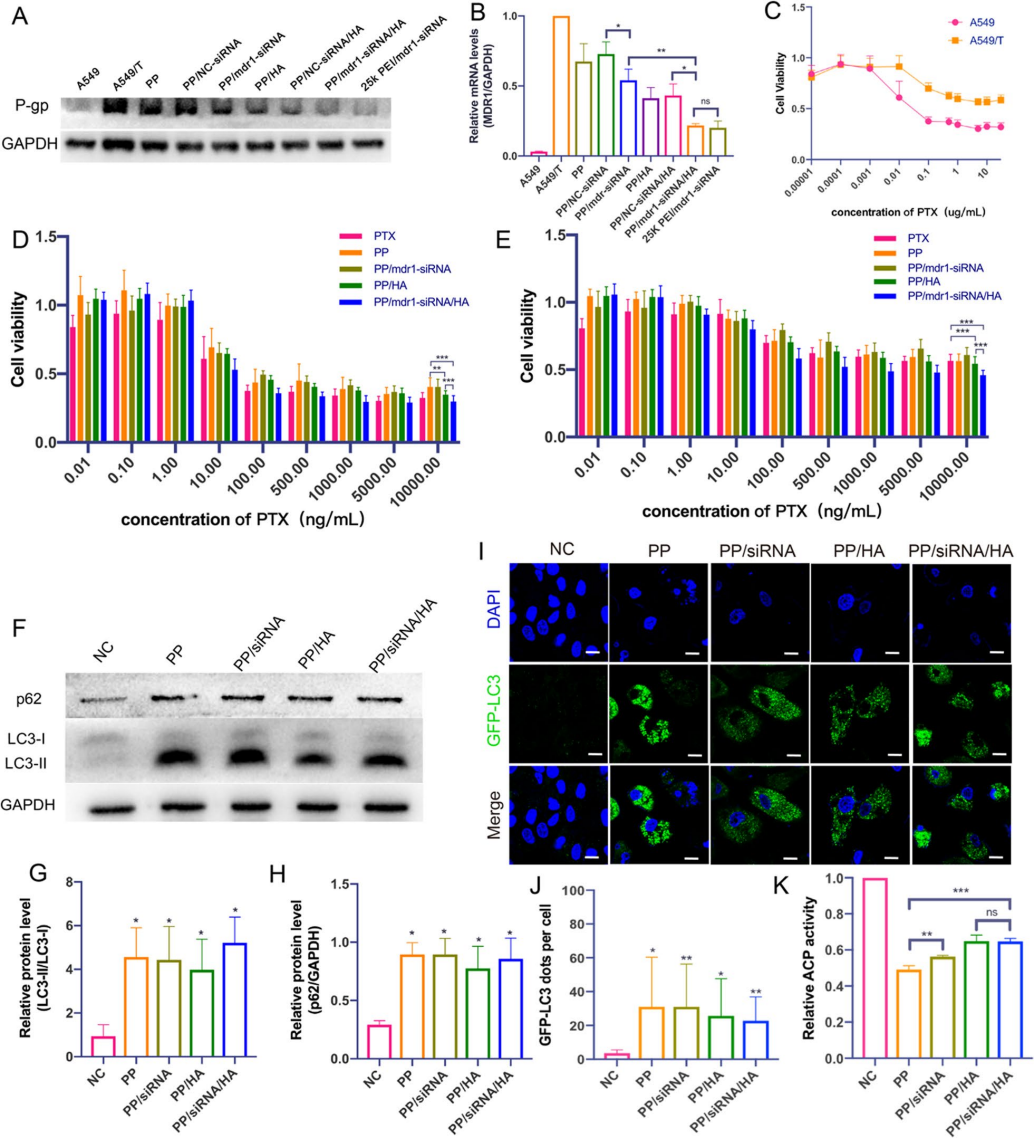
Reversing MDR of nanoassemblies (**A**) Western blot of P-gp expression in A549/T cells after treated with PP, PP/NC-siRNA, PP/mdr1-siRNA, PP/HA, PP/NC-siRNA/HA, PP/mdr1-siRNA/HA and 25 K PEI/siRNA (**B**) Relative quantification of mdr1-gene expression by q-PCR analysis. **C** Cytotoxicities of PTX in A549 and A549/T cells. Cell viability treated with various concentrations of PTX and nanoassemblies. **D** A549 cells, **E** A549/T cells. (**F**) Western blot of LC3 and p62 expression in A549/T cells after treated with nanoassemblies for 48 h. **G**, **H** Quantitative presentation of Western blotting. T test compared to NC group. **I** CLSM images of GFP-LC3 positive dots in A549T/GFP-LC3 treated with nanoassemblies for 48 h. Scale bars: 20 μm. **J** Quantified result of GFP-LC3 positive puncta. **K** The acid phosphatase activity in A549/T cells by nanoassemblies for 48 h. The data are presented as means ± SD. *p < 0.05, **p < 0.01, and ***p < 0.001

Comparing to that on A549 cells, the P-gp on the membrane of A549/T cells was significant over-expressed (Fig. 4A). 25 K *b*-PEI, as reported, was always used as positive control on account of its excellent transfection efficiency [48]. After treated with PP/siRNA and PP/siRNA/HA, the amount of P-gp on A549/T cells was significantly reduced. The silence efficiency was confirmed by quantitative PCR (Q-PCR) analysis of transfected A549/T cells, the expressions of mdr1 were reduced to 54% and 21% (Fig. 4A and B). There is no significant difference in gene silencing between PP/siRNA/HA and 25 K PEI. However, it was found that both PP and PP/HA, without siRNA-loading, showed the suppression to mdr1 gene and decreased mdr1 expression to 67% and 41%, respectively. In addition, NC-siRNA loaded nanocomplexes (PP/NC-siRNA and PP/NC-siRNA/HA) didn’t decrease the expression of P-gp compared with PP and PP/HA, which indicated the silencing effect of mdr1 siRNA was specific. Some previous studies showed that autophagy had an important role in the development of multi-drug resistance [49]. Furthermore, some compounds like chloroquine or cationic polymers could block normal autophagy flux by alkalizing lysosomes [50]. Thus, we initially guessed that the gene suppression may be caused by autophagy modulation. In addition, it could be explained for the instability of PP/siRNA without HA-coated that the silence efficiency was lower than PP/HA and PP/siRNA/HA.

### Cytotoxicity and MDR-reversing of nanoparticles

The cytotoxicity levels of different PTX formulations on A549 and A549/T cells were determined by MTT assay [51]. A549/T cells were selected to study MDR-reversing because it could overexpress P-gp, as mentioned above, and exhibited resistance to PTX (Fig. 4C). LMW PEI had the advantage of low-cytotoxicity [52], 1.8 K PEI showed low cytotoxicity levels in both A549 cells and A549/T cells (Additional file 1: Figure S16). As shown in Fig. 4D and E, all different PTX formulations could reduce the viability of A549 and A549/T cells and exhibit concentration-dependent cytotoxic effects. The results showed both PTX formulations unfolded excellent anticancer effects on A549 cells. The inhibition ratios against A549/T cells were evidently lower than that on A549 because of the drug-resistance of A549/T cells. More important, PP/siRNA/HA could more effectively enhance the inhibition ratio of A549/T cells than other PTX formulations. Notably, the excellent anticancer effect of PP/siRNA/HA may suggest gene silencing the cytotoxicity levels of different formulations on A549/T cells having relationship with the suppression of P-gp. Although PP could decrease the expression of P-gp, the inhibition ratio of it was lower than free PTX, which may be interpreted as the nanostructure disturbed by rich serum environment [41].

The half maximal inhibitory concentration (IC_50_), lower IC_50_ representing higher cytotoxicity, was further used to verify the cytotoxicity of the different formulations against cancer cells. Meanwhile, the IC_50_ value is one of the most important indexes about drug resistance of cancer cells [9]. The IC_50_ values of different PTX formulations against A549 and A549/T cells were listed in Table 2. The IC_50_ of free PTX on A549/T cells (5.5 ± 0.5 ng/mL) was 16.5-fold over that on A549 cells (90.6 ± 14.0 ng/mL), indicating the A549/T cells had acquired a great drug-resistance. The IC_50_ values to A549 cells were similar and slightly higher than that of free PTX, respectively. This was because active ingredient (PTX) needed released from preparations [42]. The IC_50_ of PP, PP/siRNA, PP/HA, and PP/siRNA/HA against A549/T cells were 35.5 ± 14.6 ng/mL, 77.5 ± 10.4 ng/mL, 31.5 ± 7.4 ng/mL, and 15.4 ± 3.0 ng/mL, respectively. PP/siRNA/HA exhibited better cytotoxicity and reversal of drug resistance on A549/T cells than other formulations.

**Table 2:** IC_50_ (of PTX) values of free PTX, PP, PP/mdr1-siRNA, PP/HA, and PP/mdr1-siRNA/HA against A549 and A549/T cells for 48 h (n = 3)

Groups	IC_50_ of PTX (ng/mL)	
	A549	A549/T
PTX	5.5 ± 0.47	90.6 ± 14.00
PP	5.4 ± 0.79	35.5 ± 14.56
PP/mdr1-siRNA	9.9 ± 1.67	77.5 ± 10.43
PP/HA	7.3 ± 0.83	31.5 ± 7.41
PP/mdr1-siRNA/HA	7.6 ± 1.69	15.4 ± 2.99

### Cell apoptosis and cell cycle

To further confirm the therapeutic effect of different PTX formulations (containing 10 ng/mL of PTX) on A549 and A549/T cells, the cell apoptosis by Annexin V-FITC/PI staining was performed at the same concentrations of 10 ng/mL of PTX. As displayed in Additional file 1: Figure S17A, both PTX formulations significantly induced late apoptosis against A549 cells. However, for A549/T cells, free PTX could not induce significant apoptosis (Additional file 1: Figure S17B). PP/siRNA/HA induced the highest apoptosis rate (87.9%) against A549/T cells compared with the control (6.7%), and PP/HA could induce similar apoptosis rate (85.4%). Notably, PTX formulations mainly induced early apoptosis against A549 cells but, to A549/T cells, mainly late apoptosis. This may result from the higher tolerance of A549/T cells. As shown in Additional file 1: Figure S18A and B, PTX could tend to arrest A549 cells in G2/M phase. However, the cell cycle of A549/T cells was not significantly different between PTX and controls, further indicating the drug resistance of A549/T cells. PP/siRNA/HA could reduce G0/G1 phase (from 65.7 to 34.7%) and tend to arrest in S phase (Additional file 1: Figure S18C), suggesting the function mechanisms of PP/siRNA/HA nanocomplex working on A549/T cells might be different from that of PTX because of autophagy inhibition. These results confirmed the ability of PP/siRNA/HA to reverse drug resistance.

### Autophagy modulation study

The gene silences experiments showed that both PP and PP/HA, without siRNA-loading, could suppress mdr1 gene expression (Fig. 4A and B). To better understand the reason that PP and PP/HA (containing 2 μM of PP) decreased mdr1 expression, the LC3/P62 expressions and numbers of autophag/autolysosomes were used to evaluating their autophagy-modulated effects. The symbol of autophagy flux initiation is the conversion of LC3B-I into LC3B-II, LC3B-I is free in cytoplasm but LC3B-II is directly binding to autophagosomes membranes [53]. Meanwhile, the level of p62, as a degradation substrate of autophagy, can reflect the autophagy flux [54]. As illustrated in Fig. 4F–H, after treatment by same gene silences experiments, there were significant accumulate of LC3-II and p62 compared to control group. Subsequently, the GFP-LC3 puncta were observed to dramatic increases under CLSM (Fig. 4I and J). Our previous research shows that the abilities of lysosomal acidity and blocking autophagic flux would reflect in the decreased activity of acid phosphatase (ACP) [55]. As shown in Fig. 4K, both nanocomplexes could decrease the ACP activity. These results suggested that nanocomplexes (PP, PP/siRNA, PP/HA, and PP/siRNA/HA) could induce autophagosome accumulation and block autophagic flux. Furthermore, we preliminarily considered that PP was the main component to modulating autophagic flux.

**Fig. 5:**
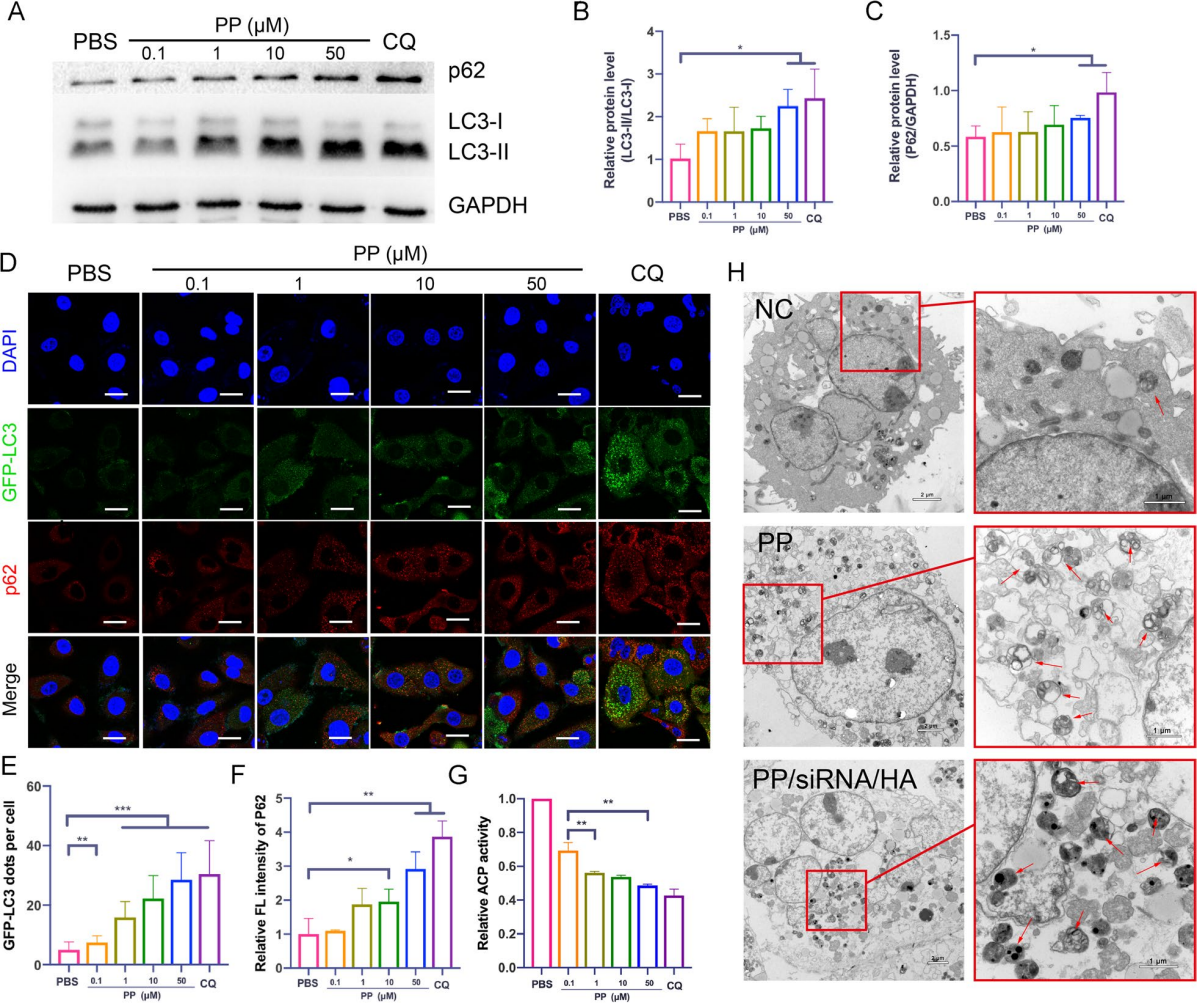
Autophagy modulation of nanoassemblies (**A**) Western blot of LC3 and p62 expression after incubated with various concentrations of PP (0–50 μM), 10 μM CQ as positive control. **B**, **C** Quantitative presentation of Western blotting. **D** CLSM images of GFP-LC3 positive dots (green) and p62 immunofluorescence staining (red) in A549T/GFP-LC3 incubated with various concentrations of PP. Cell nucleus were stained by DAPI. Scale bars: 20 μm (**E**) Quantified result of GFP-LC3 positive puncta. **F** The quantitative fluorescence intensity of p62. **G** The acid phosphatase activity after treatment of PP. **H** Bio-TEM images of A549/T cells after incubated with PP (50 μM) and PP/siRNA/HA (50 μM) for 24 h. The arrows indicate the autophagosomes. The data are presented as means ± SD. *p < 0.05, **p < 0.01, and ***p < 0.001

To further verify the autophagy modulation of PP, the markers of autophagy were assessed by western blotting and CMSL. CQ, as a common autophagy inhibitor, can block the fusion of autophagosomes via alkalizing lysosomes [56]. In our study, CQ (10 µM) would be acted as positive control of autophagy inhibition. After treated with PP for 24 h, LC3-II/LC3-I ratio showed increased and caused the accumulation of p62 (Fig. 5A–C). As showed in Fig. 5D and E, the GFP-LC3 dots per cell were increased from 7.4 to 28.5 as the concentration increase from 0.1 to 50 µM PP. The relative fluorescence intensities of p62 also were increased from 1.1 to 2.9 (Fig. 5F). The results of relative ACP activity confirmed that PP could alkaline lysosome and block degradation of autophagic substrates (Fig. 5G). TEM is an important method to observe the ultrastructural features of autophagosomes [57]. Bio-TEM images showed that upon comparisons with the control, many small double/multi-membrane vesicles and huge vacuoles were observed after treatment of 100 µM PP or PP/siRNA/HA for 24 h (Fig. 5H). These results demonstrated that high dose of PP could block autophagic flux via alkalizing lysosomes.

### In Vivo biodistribution

**Fig. 6:**
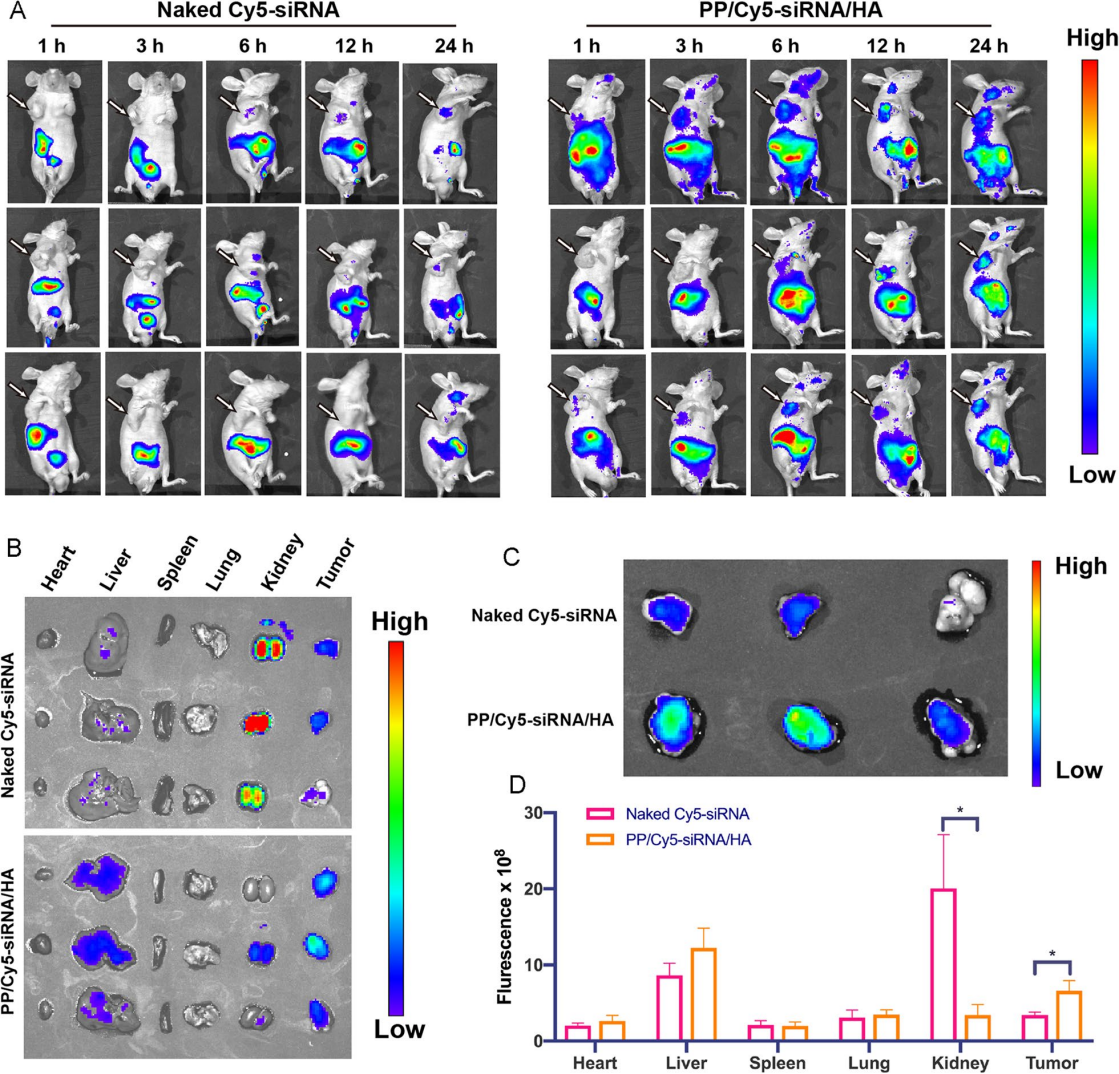
In vivo biodistribution of the PP/Cy5-siRNA/HA (1 mg/kg of Cy5-siRNA). **A** Images of whole-body after intravenous administration of Naked Cy5-siRNA or PP/Cy5-siRNA/HA in mice. The arrows indicate the tumor location. **B** Images of extracted main organs. **C** Images of extracted tumors alone. **D** Fluorescence efficiency of extracted main organs at 24 h. The data are presented as means ± SD. *p < 0.05

The bio-distribution of Cy5-siRNA loaded nanoassemblies was assessed by in vivo fluorescence imaging system after the tail vein injection of PP/Cy5-siRNA/HA in mice [58]. As shown in Fig. 6A–C, the fluorescence of naked Cy5-siRNA intensely distributed in the kidneys, but the PP/Cy5-siRNA/HA groups showed fluorescence mainly distributed in the liver, tumor, and kidneys. This could be explained by that Cy5-siRNA in solution could be excreted by the kidneys but the nanoassemblies (~ 200 nm) could be enriched in liver [59]. In addition, the fluorescence signals arising from the tumors of mice treated with PP/Cy5-siRNA/HA was 1.9-fold higher than that treated with naked Cy5-siRNA (Fig. 6D). This suggests that HA-coated nanoassemblies can not only prolong blood circulation time but also enrich at the level of tumor tissue.

### In Vivo anti-tumor effect and RNAi efficiency

**Fig. 7:**
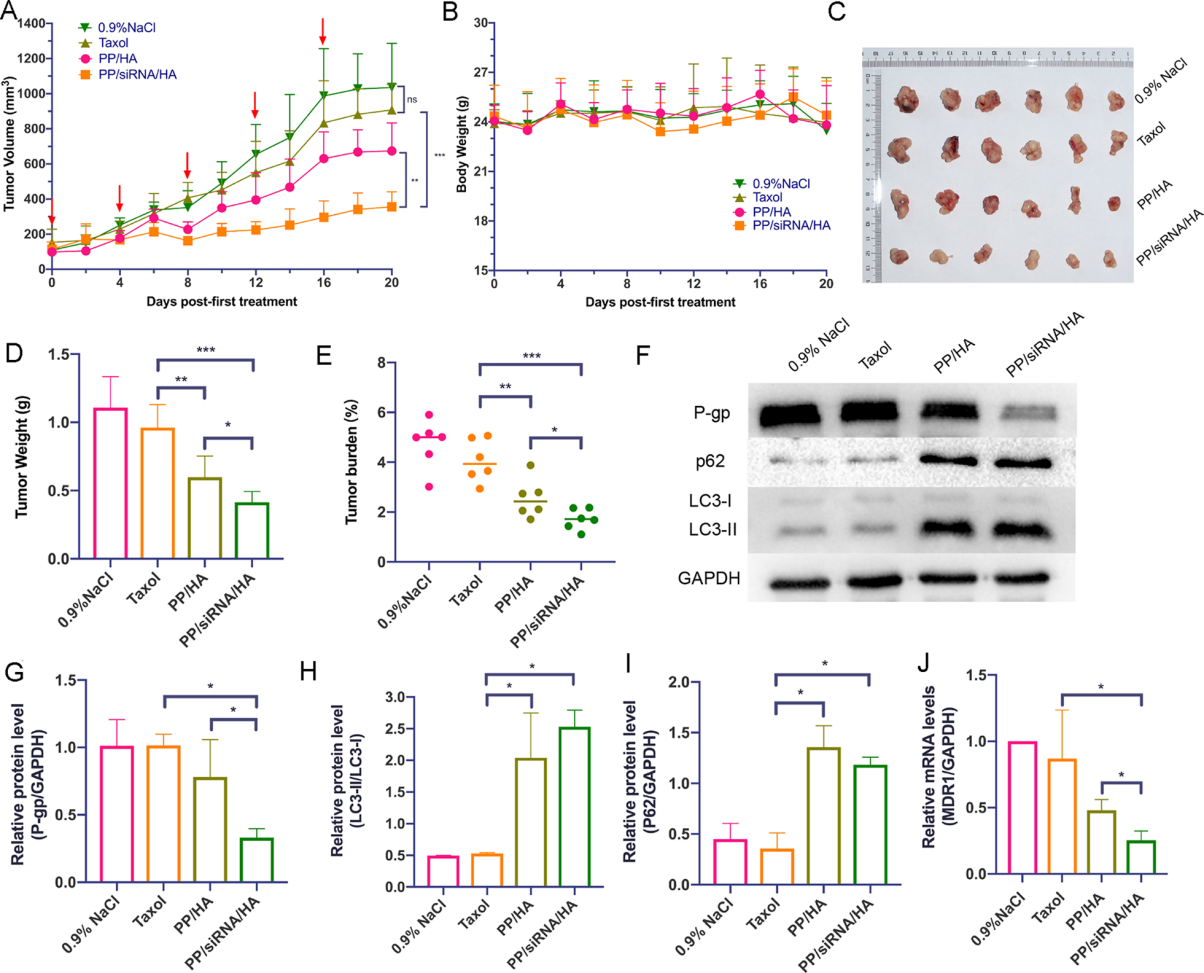
In vivo treatment efficacy of nanoassemblies against A549/T xenograft tumors (n = 6). **A** The tumor growth curves after treated with different formulations. The red arrows indicate the dates of administration. **B** Body weight changes. **C** Pictures of extracted tumors after last treatment. **D** The weights of extracted tumors. **E** Tumor burden. **F** Western blot of P-gp, p62, and LC3 expression of the tumors. **G**–**I** Quantitative presentations of Western blotting. **J** The mdr1-gene expression of the tumors by q-PCR analysis. The data are presented as means ± SD. *p < 0.05, **p < 0.01, and ***p < 0.001

**Fig. 8:**
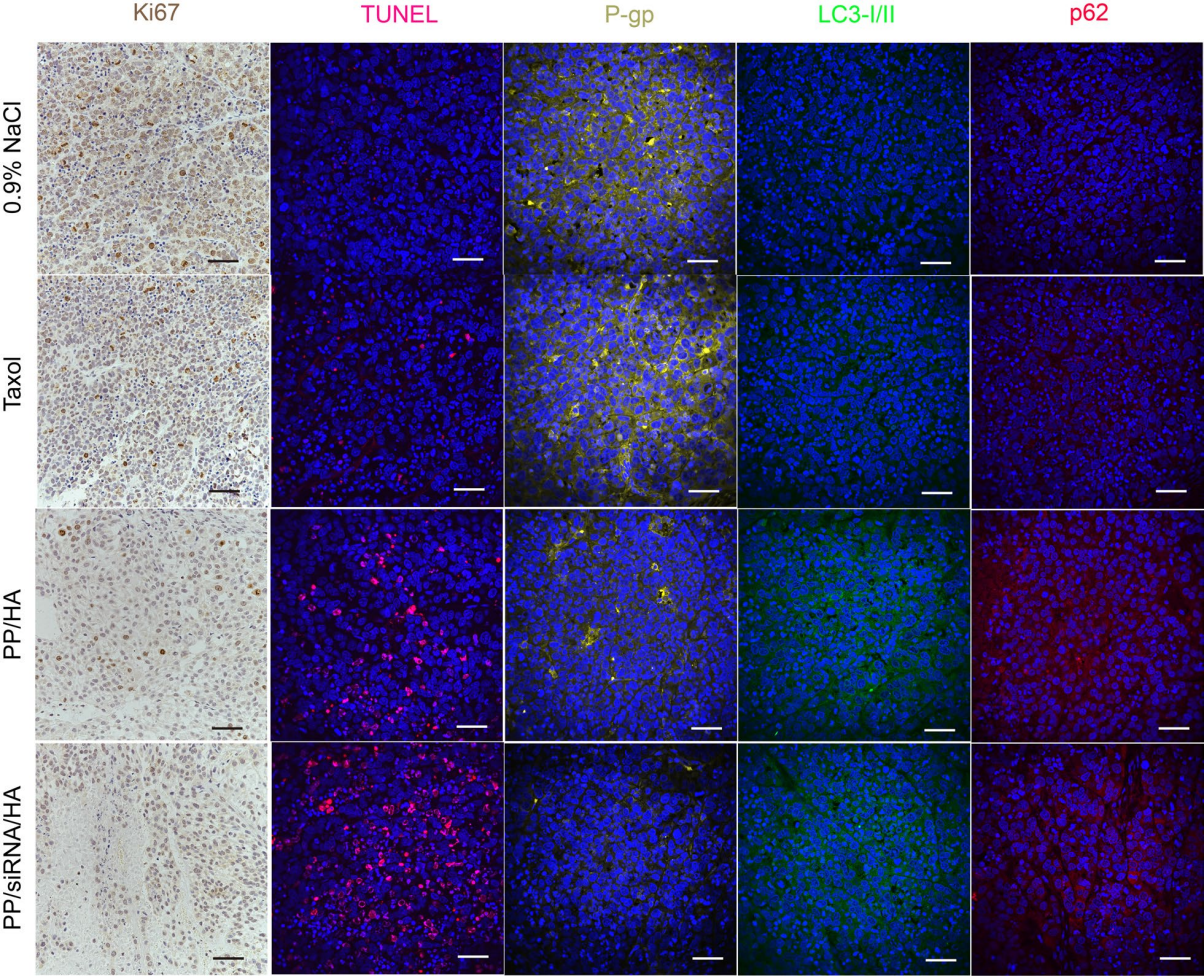
The inducing-apoptosis of nanoassemblies in tumor tissues. Immunohistochemistry assays Ki-67-positive levels and immunofluorescence staining of TUNEL-, P-gp-, LC3-, and p62- positive levels in tumor tissues. Cell nucleus were stained by DAPI. Scale bars: 50 μm

The antitumor effect was further evaluated in A549/T tumor-bearing nude mice. As illustrated in Fig. 7A, there was no significant difference between 0.9% NaCl group and Taxol group. In comparison, PP/siRNA/HA exhibited a superior antitumor efficiency with the tumor growth inhibition rates of 36.43% (Fig. 7C–E). Compared with the 0.9% NaCl and paclitaxel groups, the PP/HA group had enhanced therapeutic effects, but was still insufficient to suppress drug-resistant tumors. The tumor tissue treated with PP/siRNA/HA exhibited a more obviously decline in P-gp protein expression (Fig. 7G) and mdr1 gene expression (Fig. 7J) compared with other groups. The TUNEL results showed that the PP/siRNA/HA effectively induced more apoptosis in the tumor compared with Taxol and PP/HA (Fig. 8). The cellular proliferation of tumor was assessed by Ki67 assay (Fig. 8). Compared with the other groups, the PP/siRNA/HA group had the least cellular proliferation and was found to have the best antitumor efficacy. The result of immunofluorescence staining further confirmed the gene silence efficiency of PP/siRNA/HA with greatly reduced P-gp expression (Fig. 8). This demonstrated the PP/siRNA/HA nanocomplexes could successfully reverse the drug-resistance of tumors, and realized tumor inhibition by gene/ drug co-delivery. The weights of all groups keep basically untouched throughout the treatment (Fig. 7B). There was no significant physiological morphology abnormality in heart, liver, spleen, lung, and kidney, confirming the safety for these samples (Additional file 1: Figure S19).

### In vivo autophagy modulation

To further confirm the autophagy blockade effect of nanocomplexes, the expression of autophagy-associated proteins (LC3 and p62) in tumor was monitored at the end of the treatments. The LC3B-I/II and p62 proteins expression in tumor tissue were evaluated by western bolting and immunofluorescence staining. As showed in Fig. 7F–I, the LC3B-I/II and p62 proteins expression of PP/HA and PP/siRNA/HA were higher than 0.9% NaCl and Taxol. Moreover, there are no significant different between PP/HA and PP/siRNA/HA in the protein expressions of LC3B-I/II and p62. However, in vivo, tumor microenvironment plays an important role in the development of tumor growth and drug-resistance [51]. Thus, compared with PP/siRNA/HA, PP/HA just played limited effect of anti-tumor in vivo. The results of immunofluorescence staining further confirmed the efficiency of blocking autophagic flux (Fig. 8).

## Conclusion

We have successfully developed PP/siRNA/HA, a gene/drug co-delivery system, for combating MDR to improve clinical chemotherapy. PP is synthesized to load PTX and condense siRNA as the core of nanoassemblies, followed by coating the HA shell. These nanoassemblies can simultaneously load siRNA and PTX, which could down-regulate P-gp and efficaciously inhibit tumor growth. Furthermore, the nanoassemblies exhibited good biocompatibility with targeting lung tumor and long blood circulation time. Unlike other gene/drug delivery systems, the PP/siRNA/HA can induce autophagosome accumulation and blocks autophagic flux, which show potentials in overcoming non-pump resistance. The work provides a simple and effective strategy to combating both pump and non-pump resistance via suppressing the drug efflux pumps and blocking autophagic flow. These results supported that PP/siRNA/HA could overcome overall MDR to improve chemotherapy in vivo.

## Supplementary Information

**Additional file 1:Figure S1.** The synthetic route of PEI-PTX polymers. (a) succinyloxide, pyridine, 25 ℃; (b) HATU, HOBt, DIPEA, 25 ℃. **Figure S2.** FT-IR spectrums of PTX and PTX-SA. **Figure S3**. ^1^H NMR spectrum of PTX (A) and PTX-SA (B) in DMSO-d6 (400 MHz). **Figure S4**. Mass spectrum of PTX-SA. HRMS (ESI): exact mass calculated for [M + K]^+^ (C_51_H_55_NO_16_) requires *m/z* 976.56, found *m/z* 976.72. **Figure S5**. FT-IR spectrums of PTX-SA, PEI, PEI-PTX, and simple mixed of PEI & PTX-SA. **Figure S6**. ^1^H NMR spectrum of PTX, PEI-PTX, and PEI (400 MHz, DMSO-d_6_). **Figure S7**. Gel Permeation Chromatography spectrums of PTX, PEI, and PEI-PTX. **Figure S8**. Determination of the critical micelle concentration (CMC) of PEI-PTX. **Figure S9**. The Entrapment efficiency of FAM-siRNA at various weight ratios of PEI-PTX and FAM-siRNA by fluorophotometer. **Figure S10**. Pictures of PP, PP/siRNA, and PP/siRNA/HA nanoassembles in PBS.** Figure S11**. (A) The siRNA protection of nanoassembles in 50% FBS at various times (0-24 h). (B) The heparin resistance ability of nanoassembles at various ratios (heparin/siRNA, IU/μg). **Figure S12**. Pictures of hemolytic toxicity study with different concentrations (50, 100, 250, 500, and 1000 μg/mL) of PP or PP/HA, 0.9% NaCl as negative control, and TritonX-100 as positive control. **Figure S13**. (A) The accumulative release of PTX from PP under different conditions of pH5.0, pH7.4, pH5.0 + Esterase, and pH7.4 + Esterase (n=3). (B) The accumulative release of PTX from PP/siRNA/HA under different conditions of pH5.0, pH7.4, and pH5.0 + HAase (n=3, *P＜0.05, ***P＜0.001). **Figure S14**. (A) Flow cytometry analysis of internalization in A549 cells after treated with naked FAM-siRNA, PP/FAM-siRNA or PP/FAM-siRNA/HA for 1h and 4 h. (B) Flow cytometry analysis of internalization in A549/T cells (n=3, ***P＜0.001). **Fig. S15** (A) The Endo/lysosomal escape of PP/FAM-siRNA/HA in A549 cells by CLSM at 1 h or 4 h after 2 h uptake. Lysosomal were stained by LysoTracker (red), colocalization analysis of FAM-siRNA and lysosome at 1 h (B) or 4 h (C) by imageJ in A549 cells. (D) The Endo/lysosomal escape of PP/FAM-siRNA/HA in A549/T cells by CLSM at 1 h or 4 h after 2 h uptake. Lysosomal were stained by LysoTracker (red). Colocalization analysis of FAM-siRNA and lysosome at 1 h (E) or 4 h (F) in A549/T cells. **Figure S16**. Cytotoxicities of PEI in A549 and A549/T cells. Cell viability treated with various concentrations of 1.8K PEI. **Figure S17**. Cell apoptosis study of PTX, PP, PP/siRNA, PP/HA, and PP/siRNA/HA towards A549 cells (A) and A549/T cells (B). **Figure S18**. The cell-cycle distribution of A549 cells (A) and A549/T cells (B), and quantitive presentation of cell-cycle (C). **Figure S19**. H&E staining of tumor tissues.

## Data Availability

All supporting data for this study are included in this published article and its additional information files. All animal experiments complied with the regulations of the Animal Experiments Ethic Committee for the care and use of research animals in Qingdao University.
