# Supplementary material for: Combination of polythyleneimine regulating autophagy prodrug and Mdr1 siRNA for tumor multidrug resistance
Source: J Nanobiotechnology. 2022 Nov 11;20:476. doi: 10.1186/s12951-022-01689-y (PMC9652912; doi:10.1186/s12951-022-01689-y)
Supplement: Supplementary file 1 — Additional file 1:Figure S1. The synthetic route of PEI-PTX polymers. (a) succinyloxide, pyridine, 25 ℃; (b) HATU, HOBt, DIPEA, 25 ℃. Figure S2. FT-IR spectrums of PTX and PTX-SA. Figure S3. 1H NMR spectrum of PTX (A) and PTX-SA (B) in DMSO-d6 (400 MHz). Figure S4. Mass spectrum of PTX-SA. HRMS (ESI): exact mass calculated for [M + K]+ (C51H55NO16) requires m/z 976.56, found m/z 976.72. Figure S5. FT-IR spectrums of PTX-SA, PEI, PEI-PTX, and simple mixed of PEI & PTX-SA. Figure S6. 1H NMR spectrum of PTX, PEI-PTX, and PEI (400 MHz, DMSO-d6). Figure S7. Gel Permeation Chromatography spectrums of PTX, PEI, and PEI-PTX. Figure S8. Determination of the critical micelle concentration (CMC) of PEI-PTX. Figure S9. The Entrapment efficiency of FAM-siRNA at various weight ratios of PEI-PTX and FAM-siRNA by fluorophotometer. Figure S10. Pictures of PP, PP/siRNA, and PP/siRNA/HA nanoassembles in PBS. Figure S11. (A) The siRNA protection of nanoassembles in 50% FBS at various times (0-24 h). (B) The heparin resistance ability of nanoassembles at various ratios (heparin/siRNA, IU/μg). Figure S12. Pictures of hemolytic toxicity study with different concentrations (50, 100, 250, 500, and 1000 μg/mL) of PP or PP/HA, 0.9% NaCl as negative control, and TritonX-100 as positive control. Figure S13. (A) The accumulative release of PTX from PP under different conditions of pH5.0, pH7.4, pH5.0 + Esterase, and pH7.4 + Esterase (n=3). (B) The accumulative release of PTX from PP/siRNA/HA under different conditions of pH5.0, pH7.4, and pH5.0 + HAase (n=3, *P＜0.05, ***P＜0.001). Figure S14. (A) Flow cytometry analysis of internalization in A549 cells after treated with naked FAM-siRNA, PP/FAM-siRNA or PP/FAM-siRNA/HA for 1h and 4 h. (B) Flow cytometry analysis of internalization in A549/T cells (n=3, ***P＜0.001). Fig. S15 (A) The Endo/lysosomal escape of PP/FAM-siRNA/HA in A549 cells by CLSM at 1 h or 4 h after 2 h uptake. Lysosomal were stained by LysoTracker (red), colocalization analysis of [file 12951_2022_1689_MOESM1_ESM.doc]

Additioal file 1


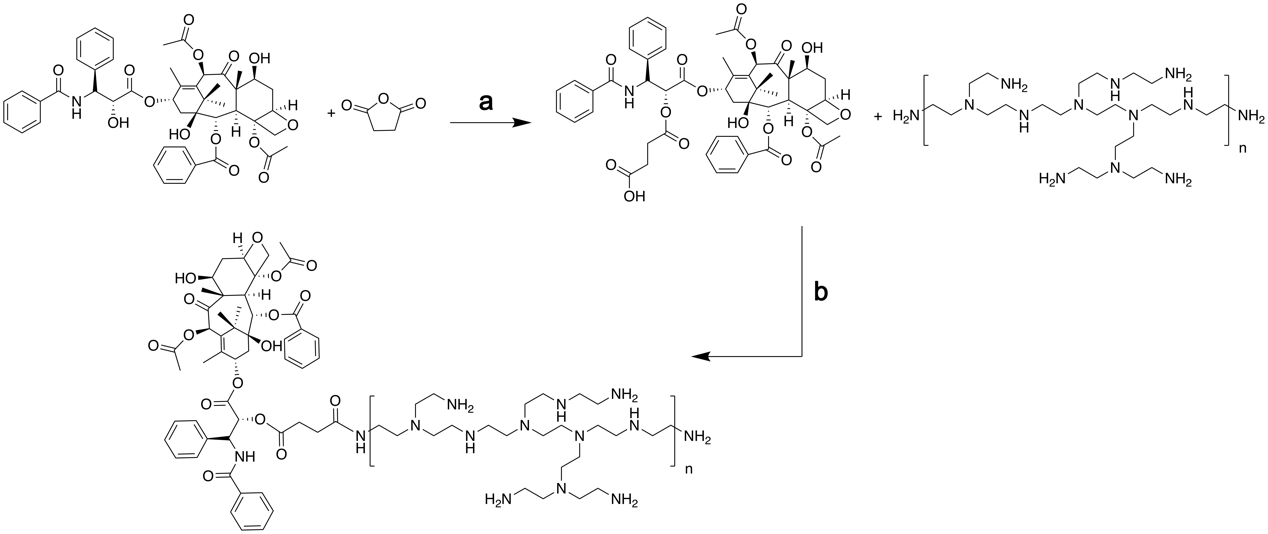


**Figure S1.** The synthetic route of PEI-PTX polymers. (a) succinyloxide, pyridine, 25 ℃; (b) HATU, HOBt, DIPEA, 25 ℃.


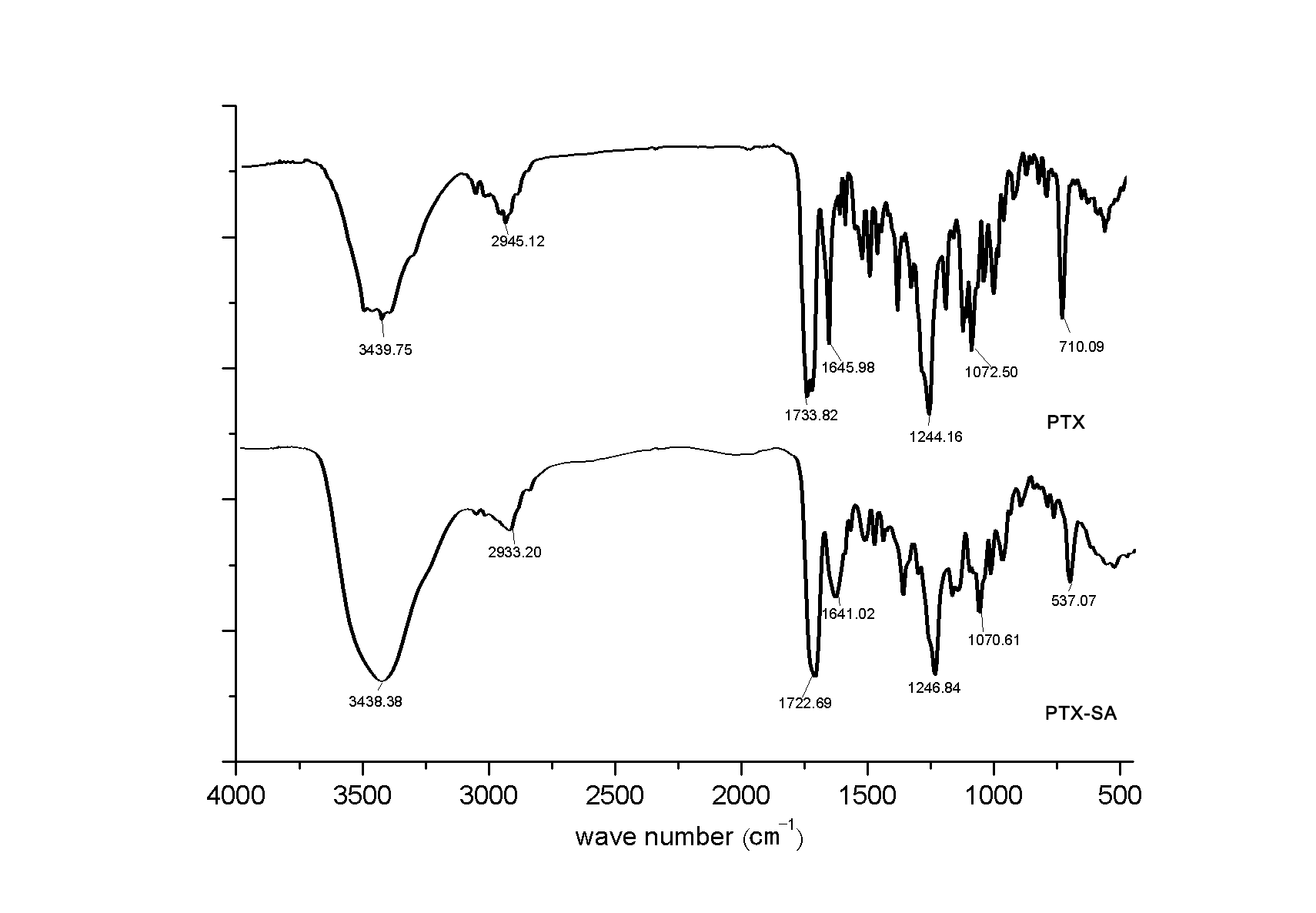


**Figure S2.** FT-IR spectrums of PTX and PTX-SA.


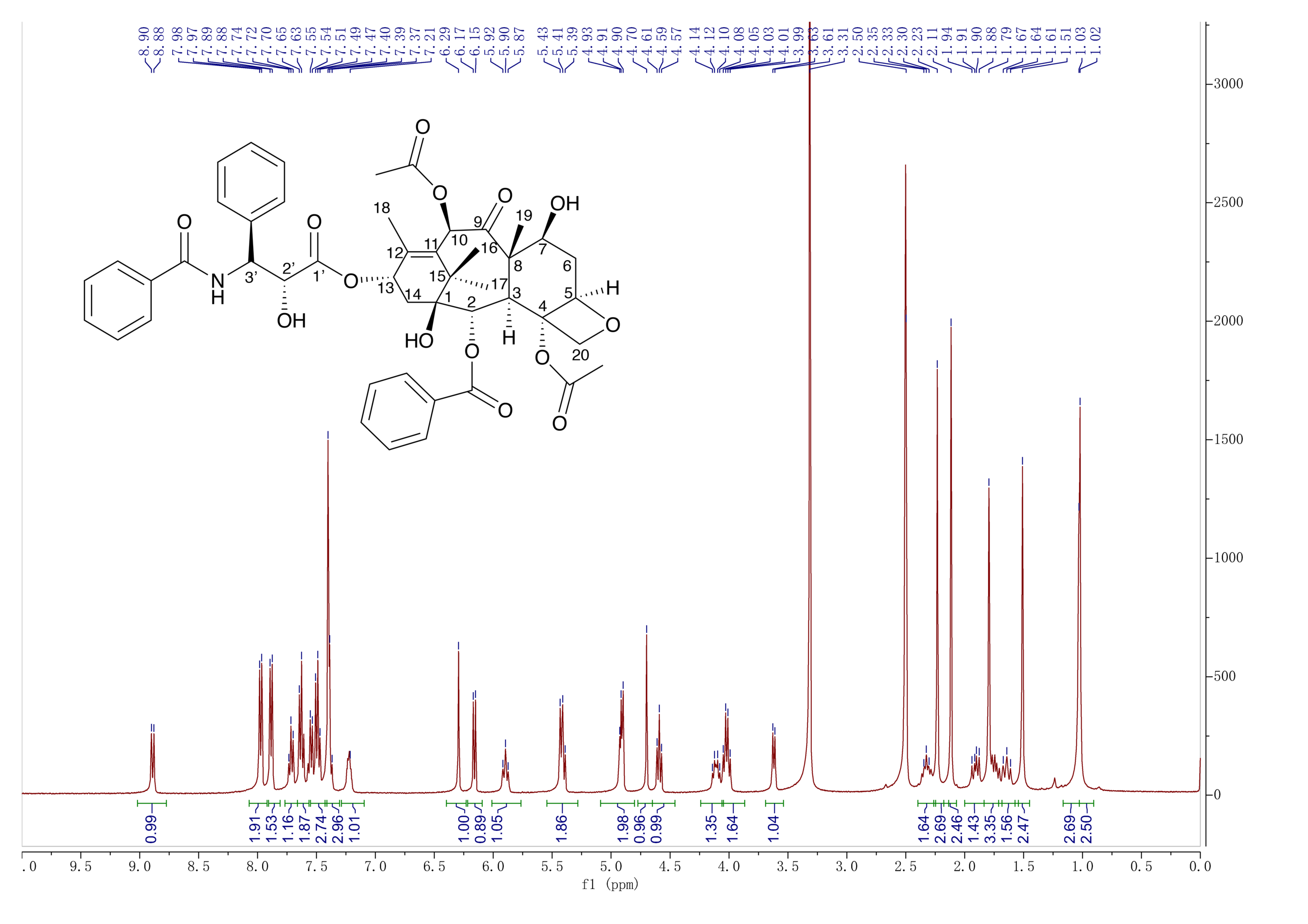

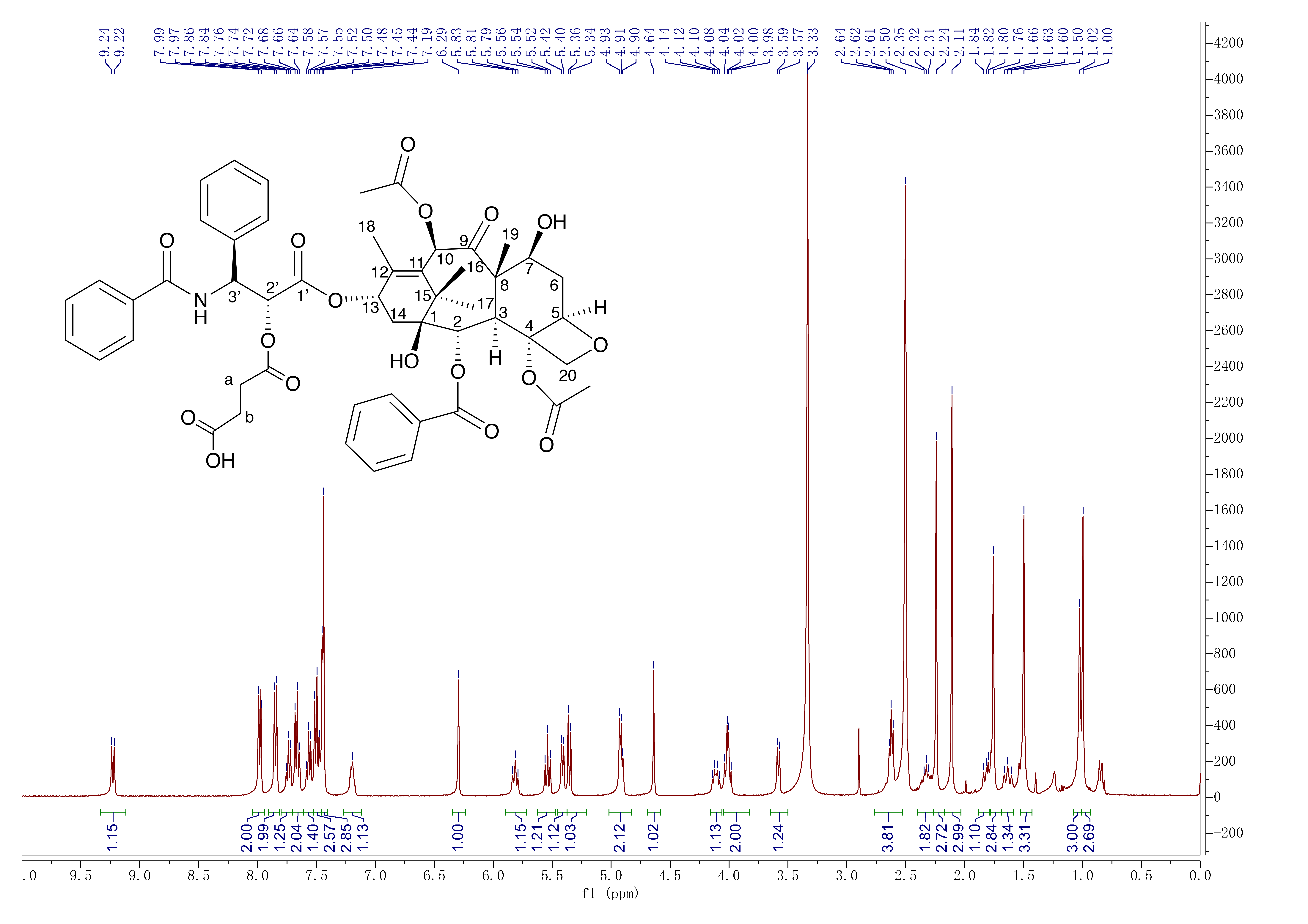


A

B

**Figure S3**. ^1^H NMR spectrum of PTX (A) and PTX-SA (B) in DMSO-d6 (400 MHz).

**Signals assignments of PTX** **spectrum: ^1^H NMR (400 MHz, DMSO-d_6_),** **δ (ppm):** 8.98 (1H, d, J = 8.6 Hz,3’-NH-), 7.98-7.21 (15H, Ar-H), 6.29 (1H, s, 10-H), 6.16 (1H, d, J = 7.6 Hz, 2’-OH), 5.90 (1H, t, J = 9.0 Hz, 13-H), 5.54-5.28 (1H, m, 3’-H), 4.93 (1H, m, 5-H), 4.70 (1H, s, 2’-H), 4.59 (1H, t, J = 7.7 Hz, 2-H), 4.24-4.05 (1H, m, 7-H), 4.06-3.87 (2H, m, 20-H), 3.62 (1H, d, J=7.2 Hz, 3-H), 2.40-2.25 (2H, m, 6, 14-αH), 2.23 (3H, s, 4-COCH_3_), 2.11 (3H, s, 10-COCH_3_), 1.91 (1H, m, 6-βH), 1.79 (3H, s, 18-H), 1.71-1.54 (1H, m, 14-H), 1.51 (3H, s, 19-H), 1.03 (3H, s, 17-H), 1.02 (3H, s, 16-H)

**Signals assignments of PTX-SA** **spectrum: ^1^H NMR (400 MHz, DMSO-d_6_), δ (ppm):** 12.28 (1H, s, b-COOH), 9.22 (1H, d, J=8.5 Hz, 3’-NH), 7.99-7.19 (15H, Ar-H), 6.29 (1H, s, 10-H), 5.81 (1H, t, J = 8.9 Hz, 13-H), 5.54 (1H, t, J = 8.7 Hz, 3’-H), 5.34 (1H, d, J = 9.0 Hz, 2-H), 4.93 (1H, m, 5-H), 4.64 (1H, s, 2’-H), 4.12 (1H, q, J = 6.8 Hz, 7-H), 4.02 (2H, q, J = 8.3 Hz, 20-H), 3.57 (1H, d, J=7.1 Hz, 3-H), 2.76-2.55 (4H, m, a, b-H), 2.41-2.26 (2H, m, 6, 14-αH), 2.24 (3H, s, 4-COCH_3_), 2.11 (3H, s, 10-COCH_3_), 1.87-1.79 (1H, m, 14-H), 1.76 (3H, s, 18-H), 1.63(1H, m, 6-βH), 1.50 (3H, s, 19-H), 1.02 (3H, s, 17-H), 1.00 (3H, s, 16-H).

**Figure S4**. Mass spectrum of PTX-SA. HRMS (ESI): exact mass calculated for [M + K]^+^ (C_51_H_55_NO_16_) requires *m/z* 976.56, found *m/z* 976.72.


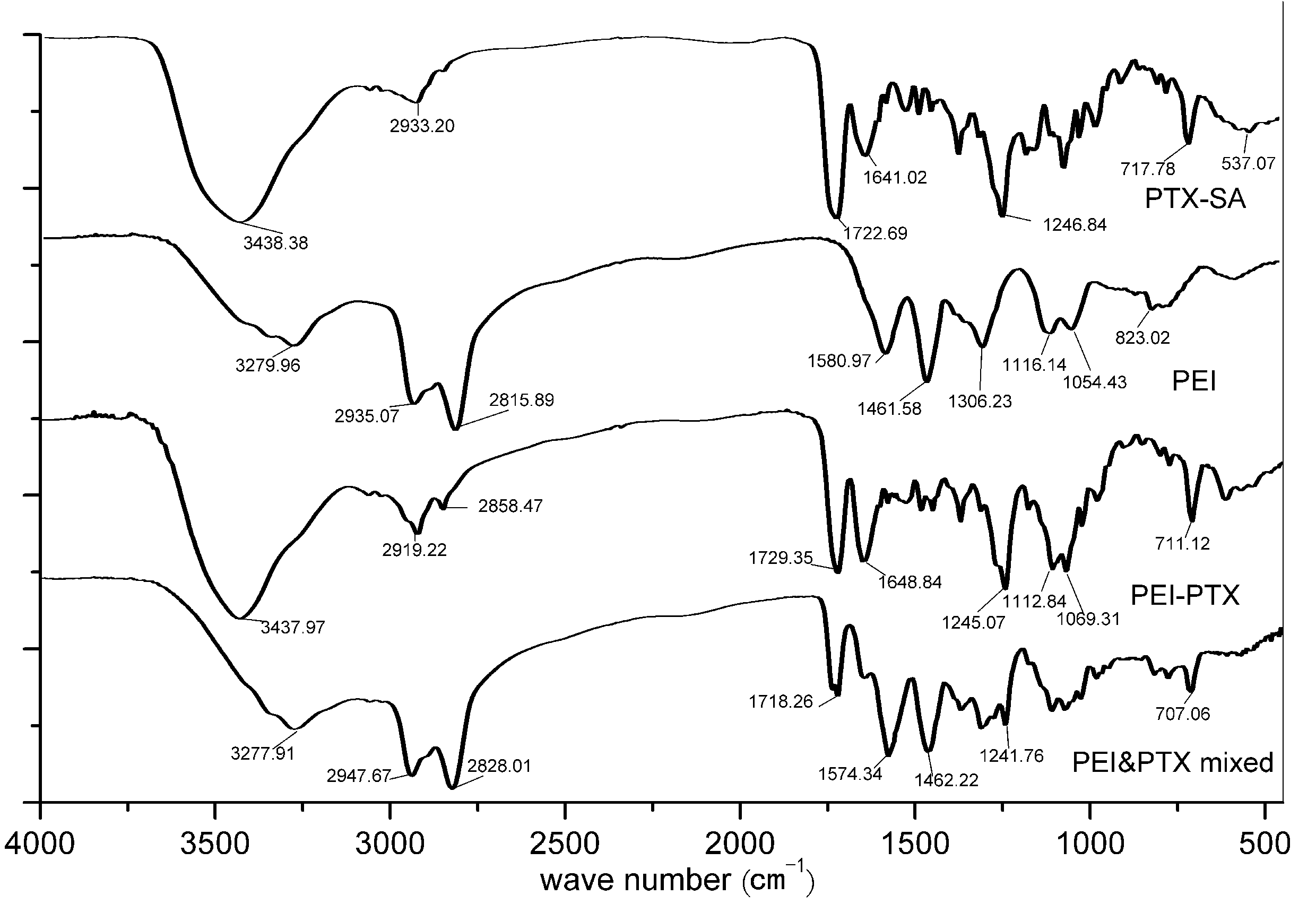


**Figure S5**. FT-IR spectrums of PTX-SA, PEI, PEI-PTX, and simple mixed of PEI & PTX-SA.


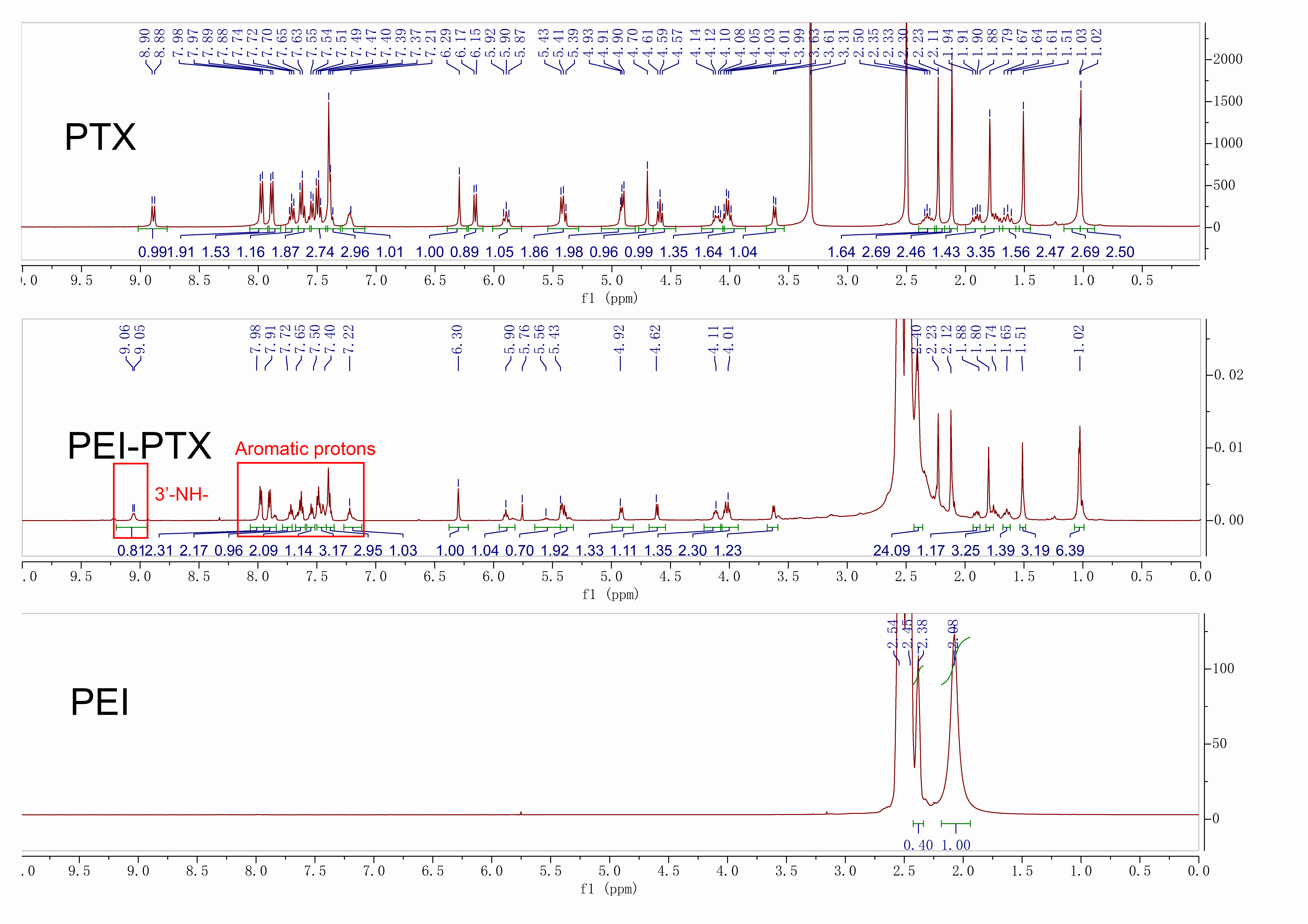


**Figure S6**. ^1^H NMR spectrum of PTX, PEI-PTX, and PEI (400 MHz, DMSO-d_6_).

The calculation method to decide the content of PTX in PP:

The δ 2.08 ppm peak in ^1^H NMR spectrum of PEI is assigned to amine protons which are 23.81% protons of total PEI’s protons, and the integral of δ 2.4 ppm peak is 0.4-times of the integral of δ2.08 ppm. In addition, due to no δ 2.4 ppm signal in the ^1^H NMR spectrum of PTX, the PTX content in PP is calculated through comparing integrals of peaks at δ 8.05-7.15 (aromatic protons of PTX) with δ 2.45-2.35 (partial methylene protons of PEI) in the ^1^H NMR spectrum of PP. The calculation formula is as follows:


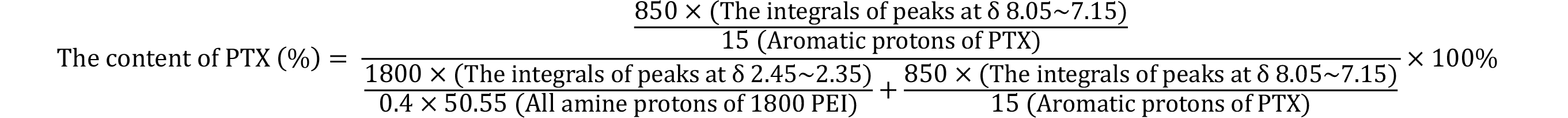


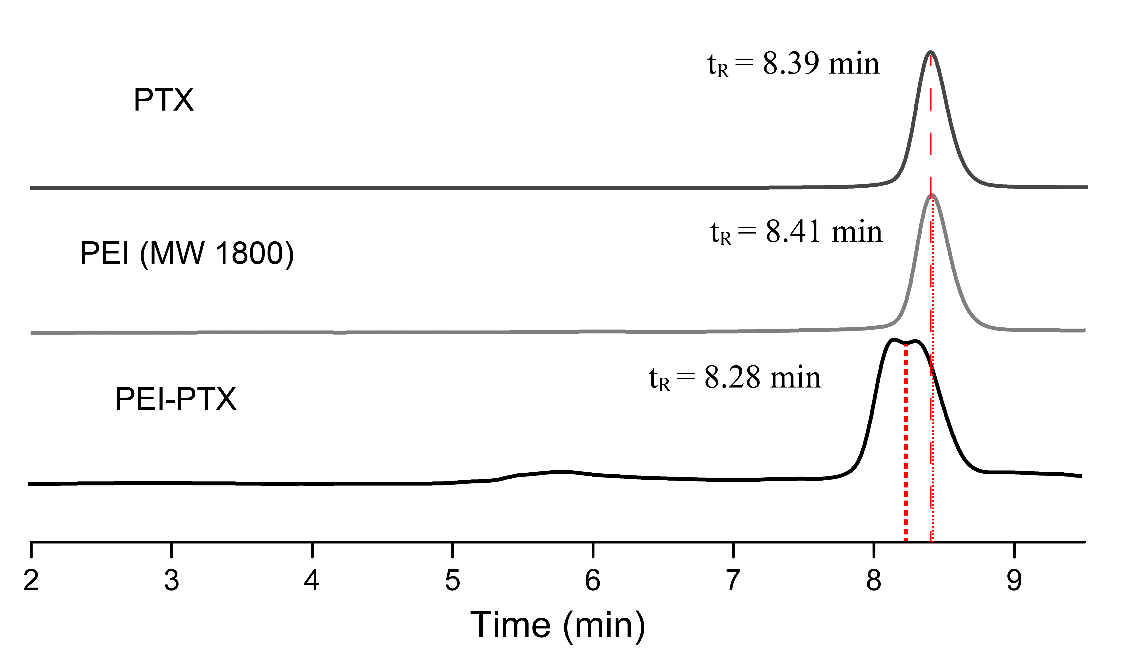


|  | Mp | Mn | Mw | PDI |
| --- | --- | --- | --- | --- |
| PEI | 1287.35 | 1291.00 | 1310.57 | 1.0152 |
| PTX | 1303.96 | 1304.84 | 1319.78 | 1.0114 |
| PEI-PTX | 1506.34 | 1380.96 | 1527.22 | 1.1059 |

**Figure S7**. Gel Permeation Chromatography spectrums of PTX, PEI, and PEI-PTX. Polystyrene as calibration for the measurements.


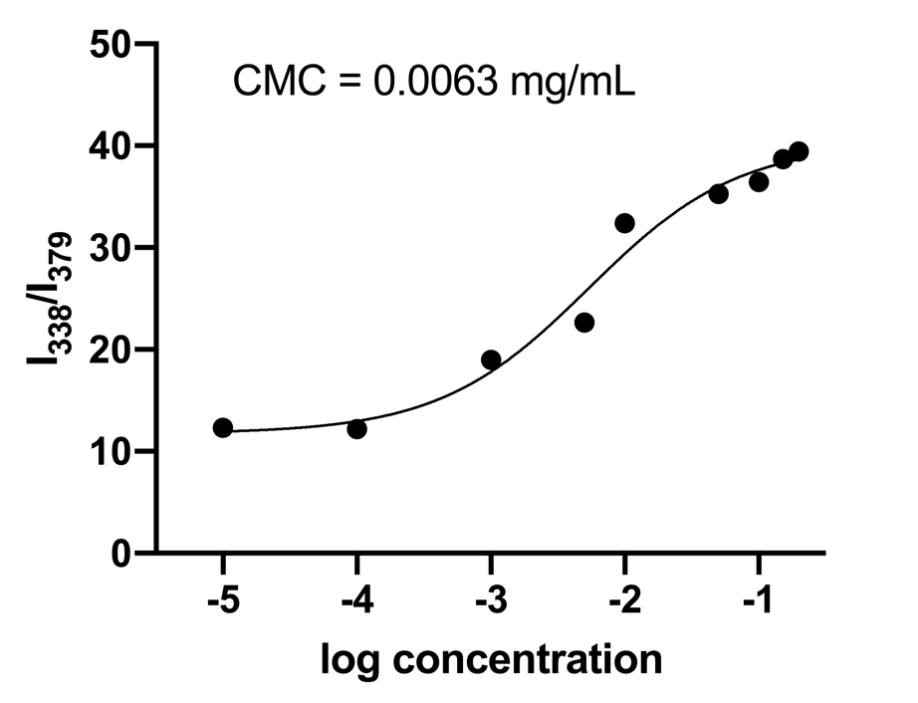


**Figure S8**. Determination of the critical micelle concentration (CMC) of PEI-PTX.

**Figure S9**. The Entrapment efficiency of FAM-siRNA at various weight ratios of PEI-PTX and FAM-siRNA by fluorophotometer.


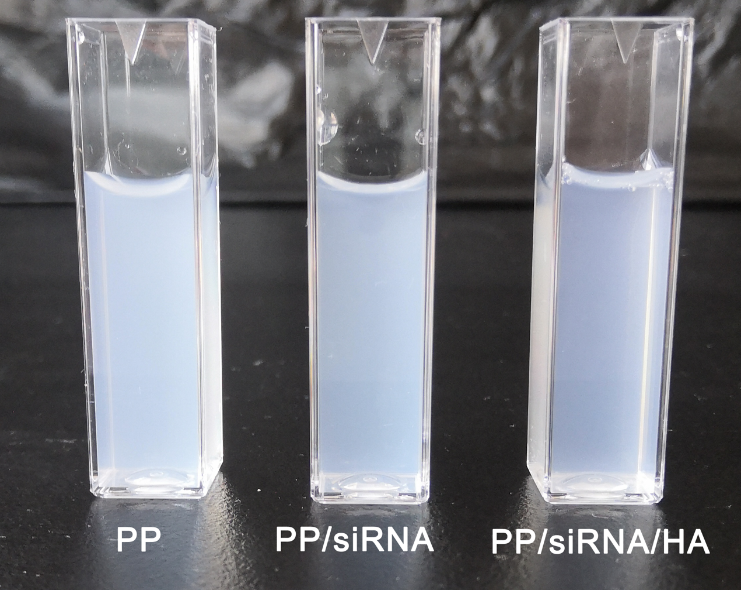


**Figure S10**. Pictures of PP, PP/siRNA, and PP/siRNA/HA nanoassembles in PBS.


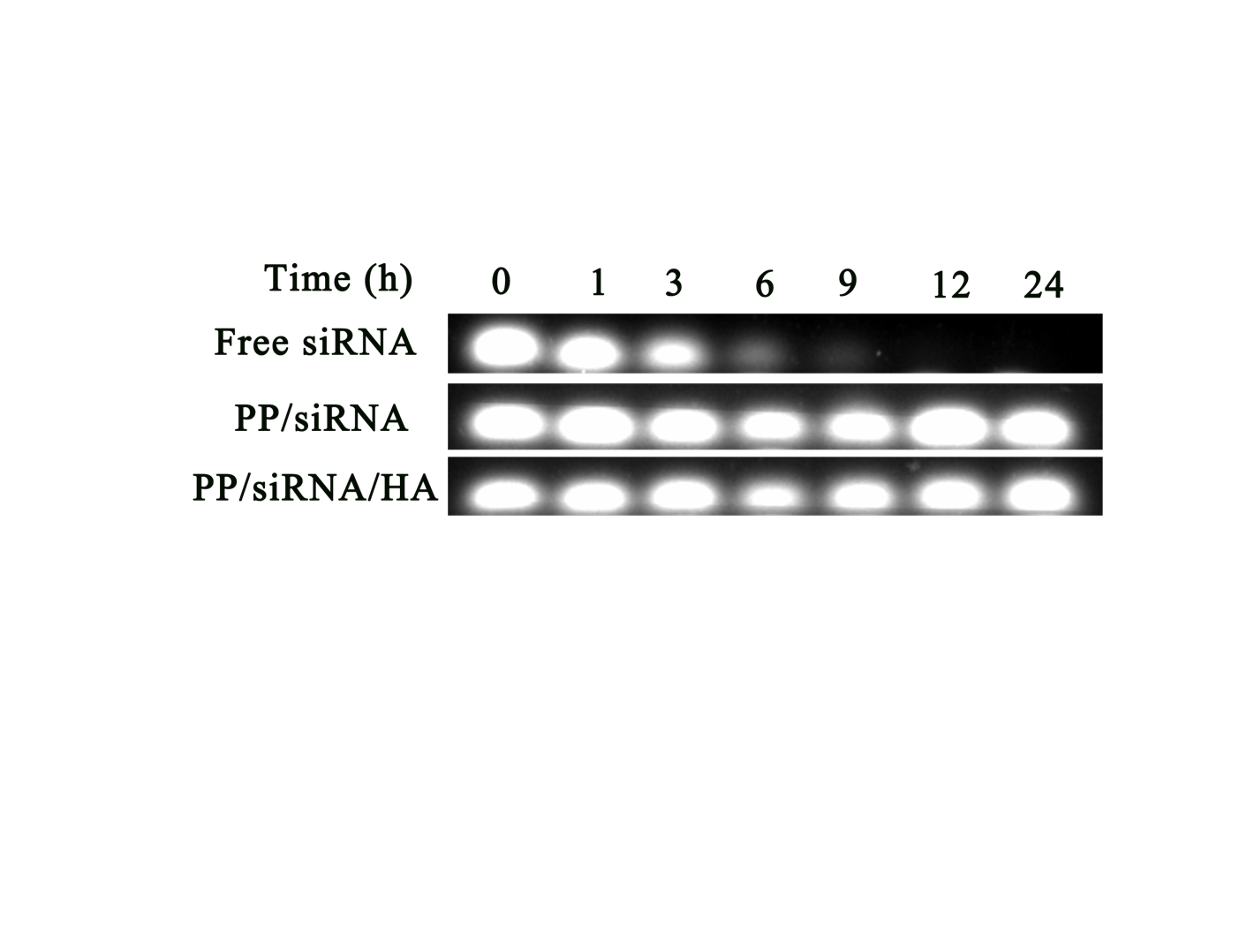

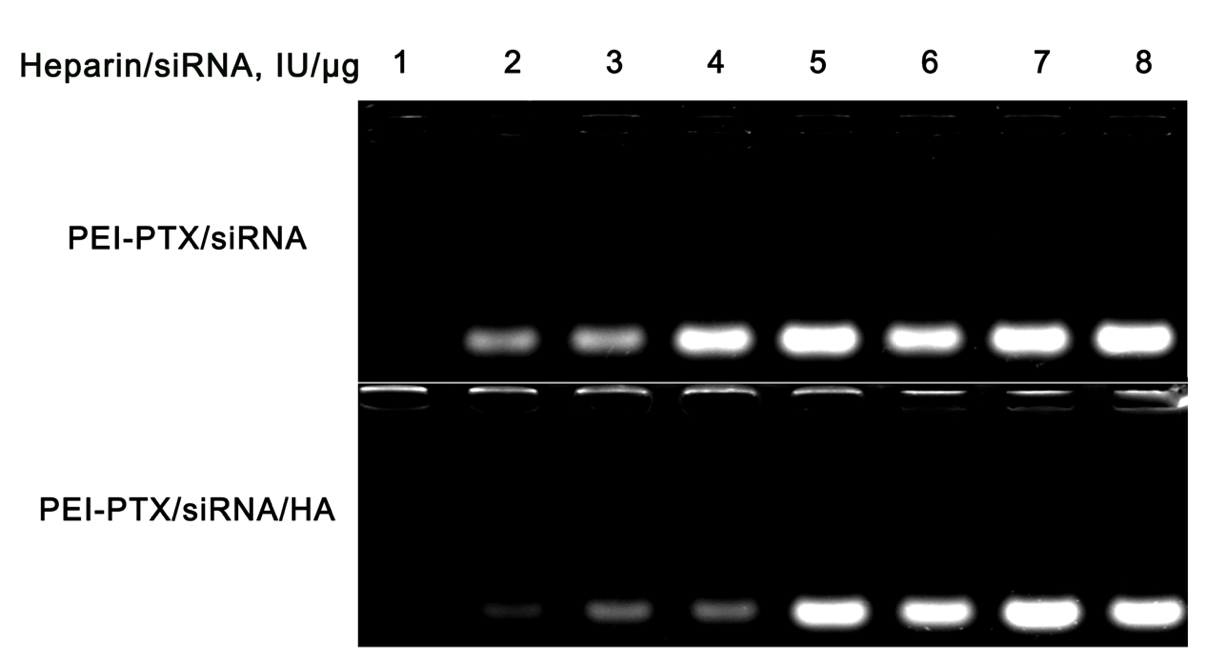


B

A

**Figure S11**. (A) The siRNA protection of nanoassembles in 50% FBS at various times (0-24 h). (B) The heparin resistance ability of nanoassembles at various ratios (heparin/siRNA, IU/μg).


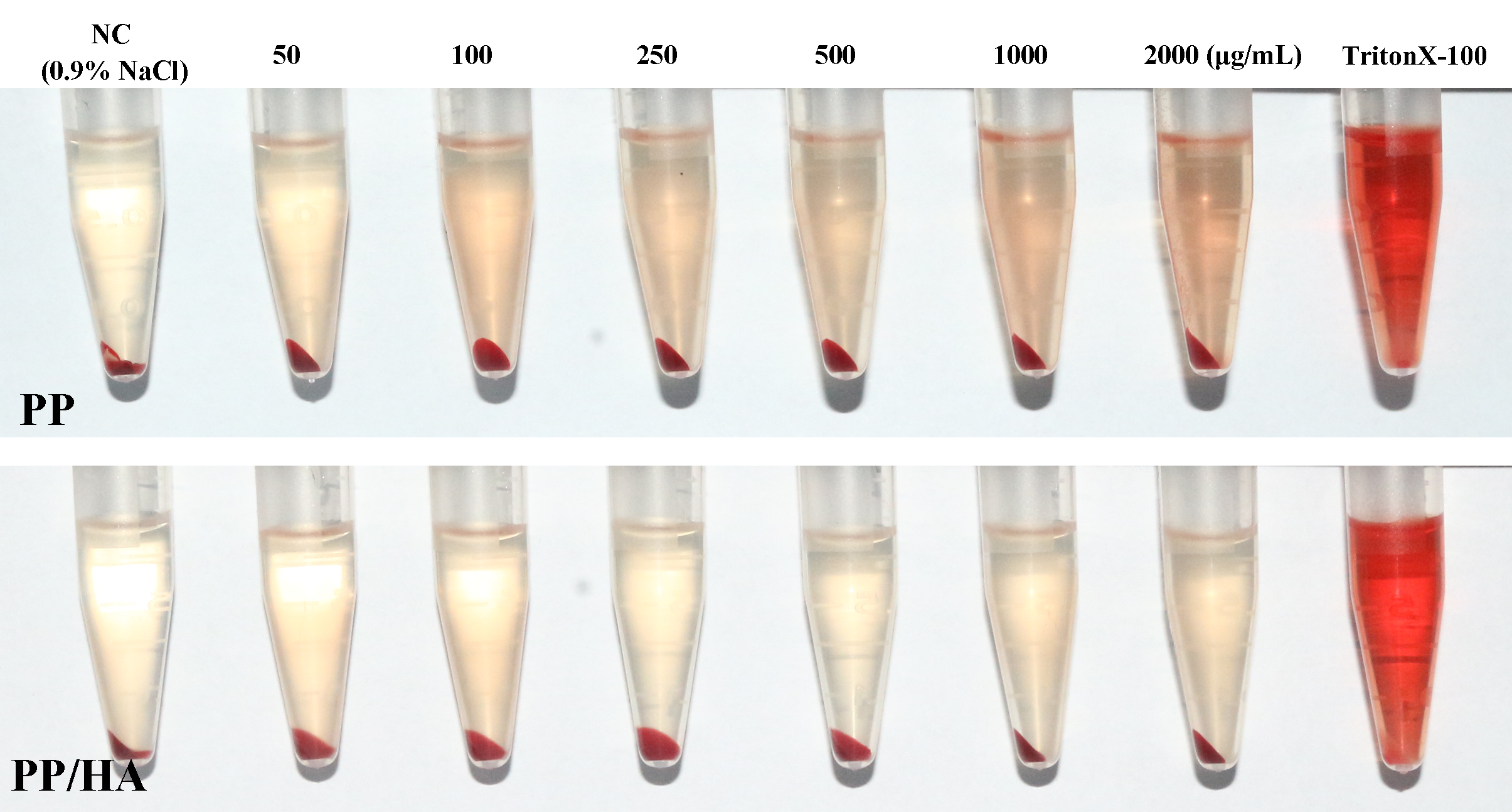


**Figure S12**. Pictures of hemolytic toxicity study with different concentrations (50, 100, 250, 500, and 1000 μg/mL) of PP or PP/HA, 0.9% NaCl as negative control, and TritonX-100 as positive control.


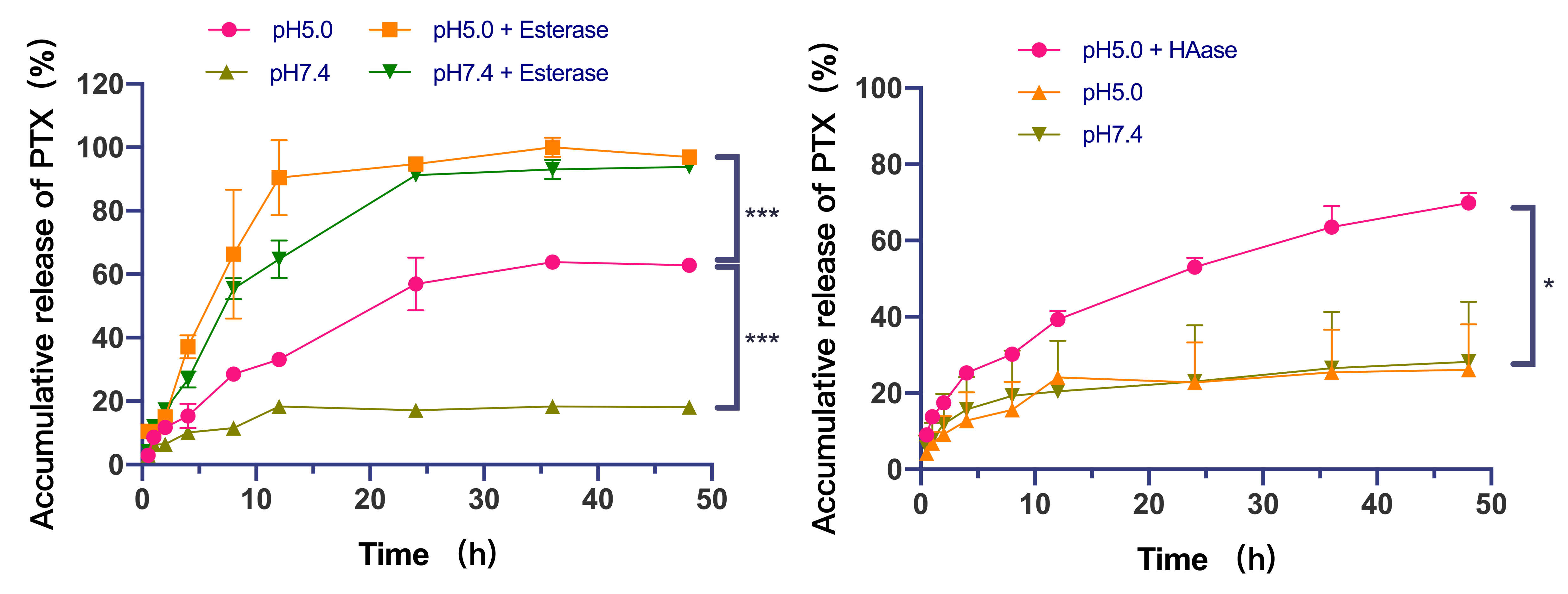


A

B

**Figure S13**. (A) The accumulative release of PTX from PP under different conditions of pH5.0, pH7.4, pH5.0 + Esterase, and pH7.4 + Esterase (n=3). (B) The accumulative release of PTX from PP/siRNA/HA under different conditions of pH5.0, pH7.4, and pH5.0 + HAase (n=3, *P＜0.05, ***P＜0.001).


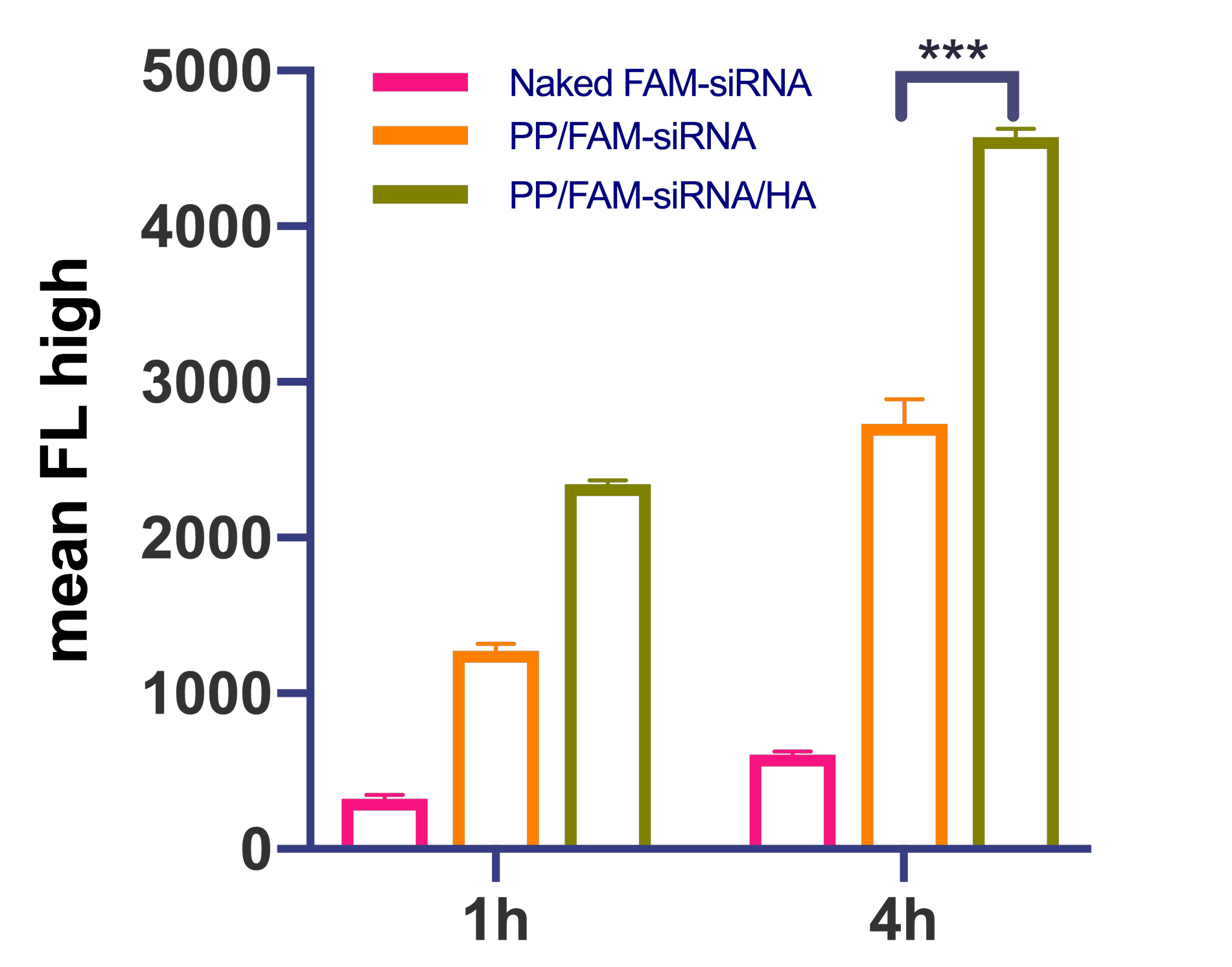

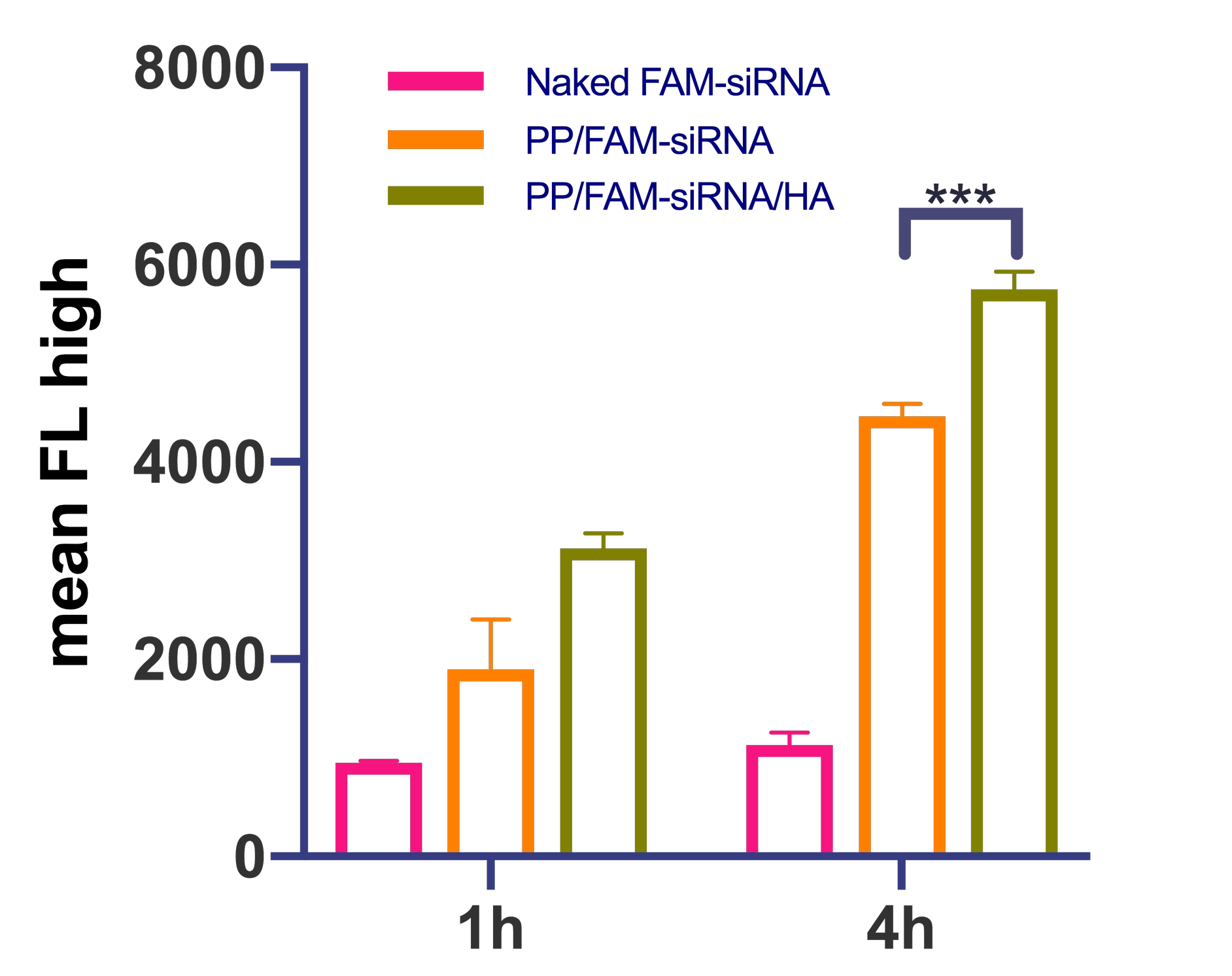


A

B

**Figure S14**. (A) Flow cytometry analysis of internalization in A549 cells after treated with naked FAM-siRNA, PP/FAM-siRNA or PP/FAM-siRNA/HA for 1h and 4 h. (B) Flow cytometry analysis of internalization in A549/T cells (n=3, ***P＜0.001).


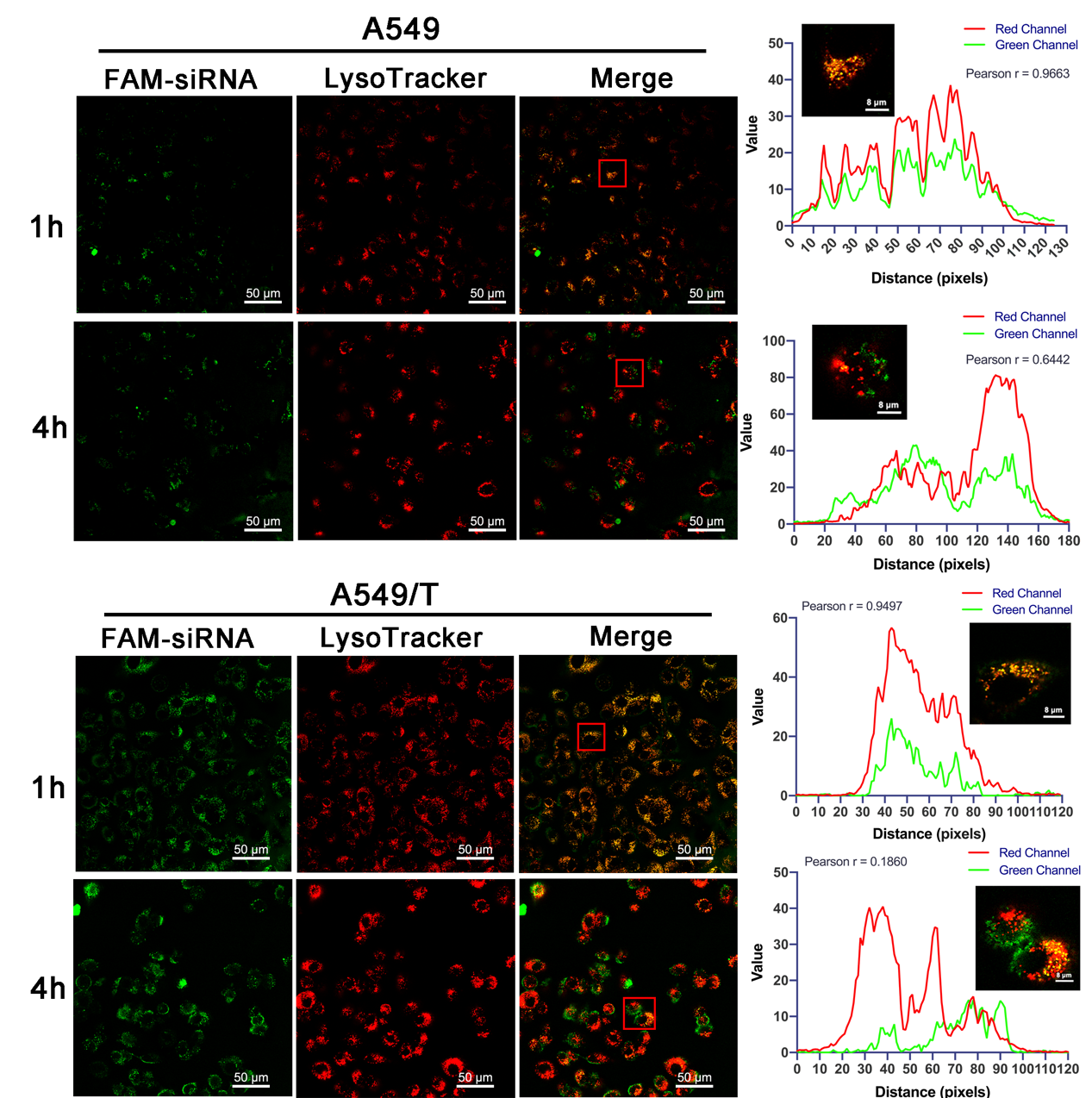


A

B

C

D

E

F

**Fig. S15** (A) The Endo/lysosomal escape of PP/FAM-siRNA/HA in A549 cells by CLSM at 1 h or 4 h after 2 h uptake. Lysosomal were stained by LysoTracker (red), colocalization analysis of FAM-siRNA and lysosome at 1 h (B) or 4 h (C) by imageJ in A549 cells. (D) The Endo/lysosomal escape of PP/FAM-siRNA/HA in A549/T cells by CLSM at 1 h or 4 h after 2 h uptake. Lysosomal were stained by LysoTracker (red). Colocalization analysis of FAM-siRNA and lysosome at 1 h (E) or 4 h (F) in A549/T cells.


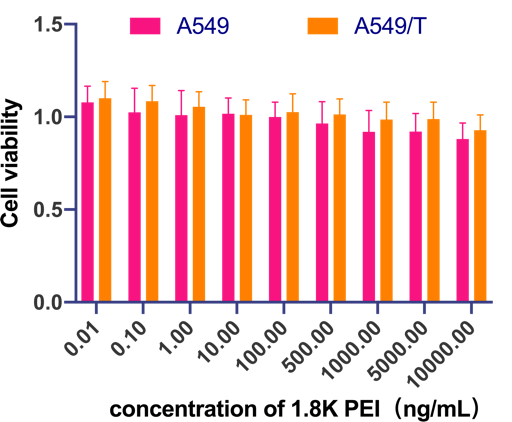


**Figure S16**. Cytotoxicities of PEI in A549 and A549/T cells. Cell viability treated with various concentrations of 1.8K PEI.


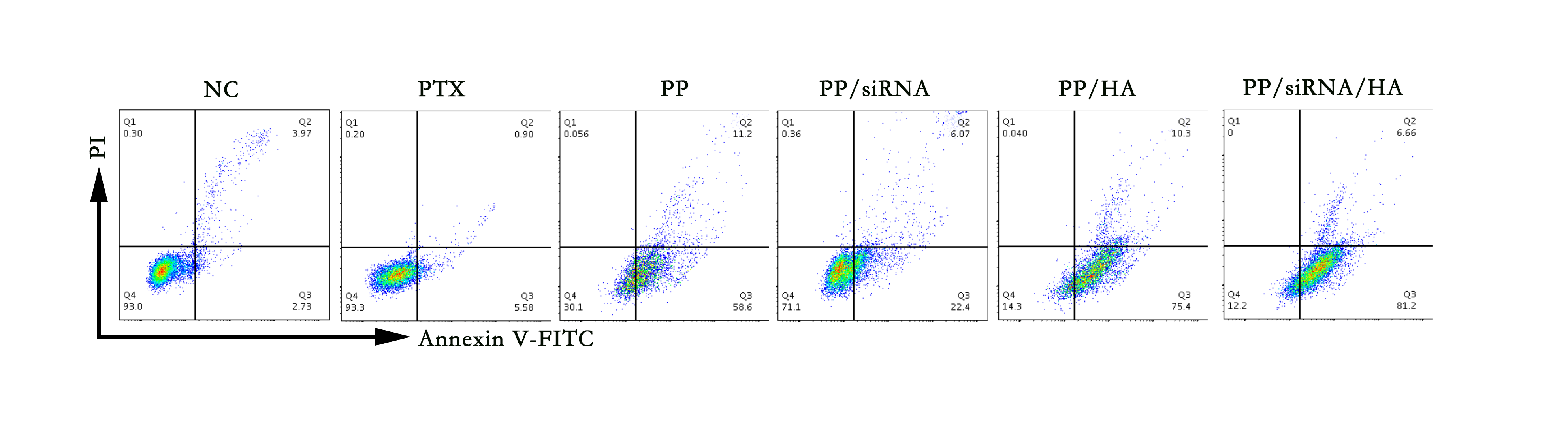

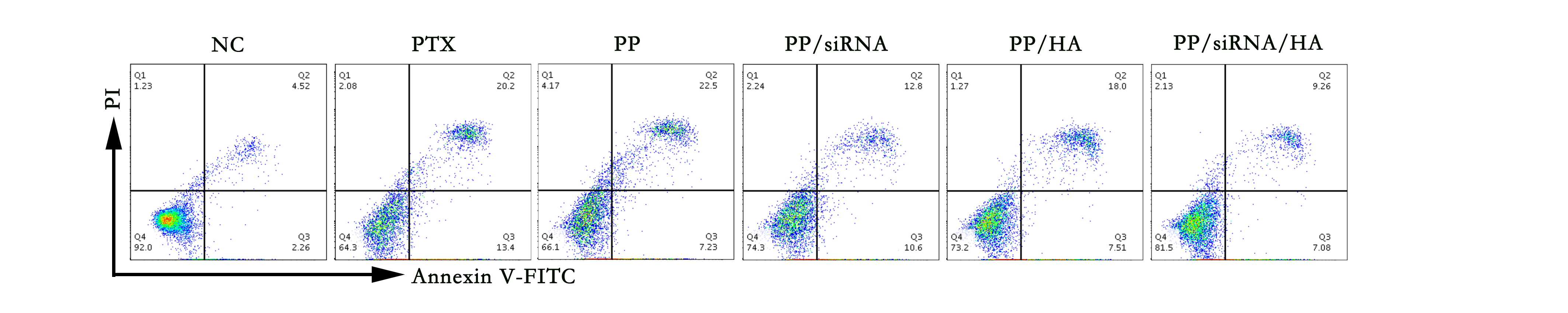


A

B

**Figure S17**. Cell apoptosis study of PTX, PP, PP/siRNA, PP/HA, and PP/siRNA/HA towards A549 cells (A) and A549/T cells (B).


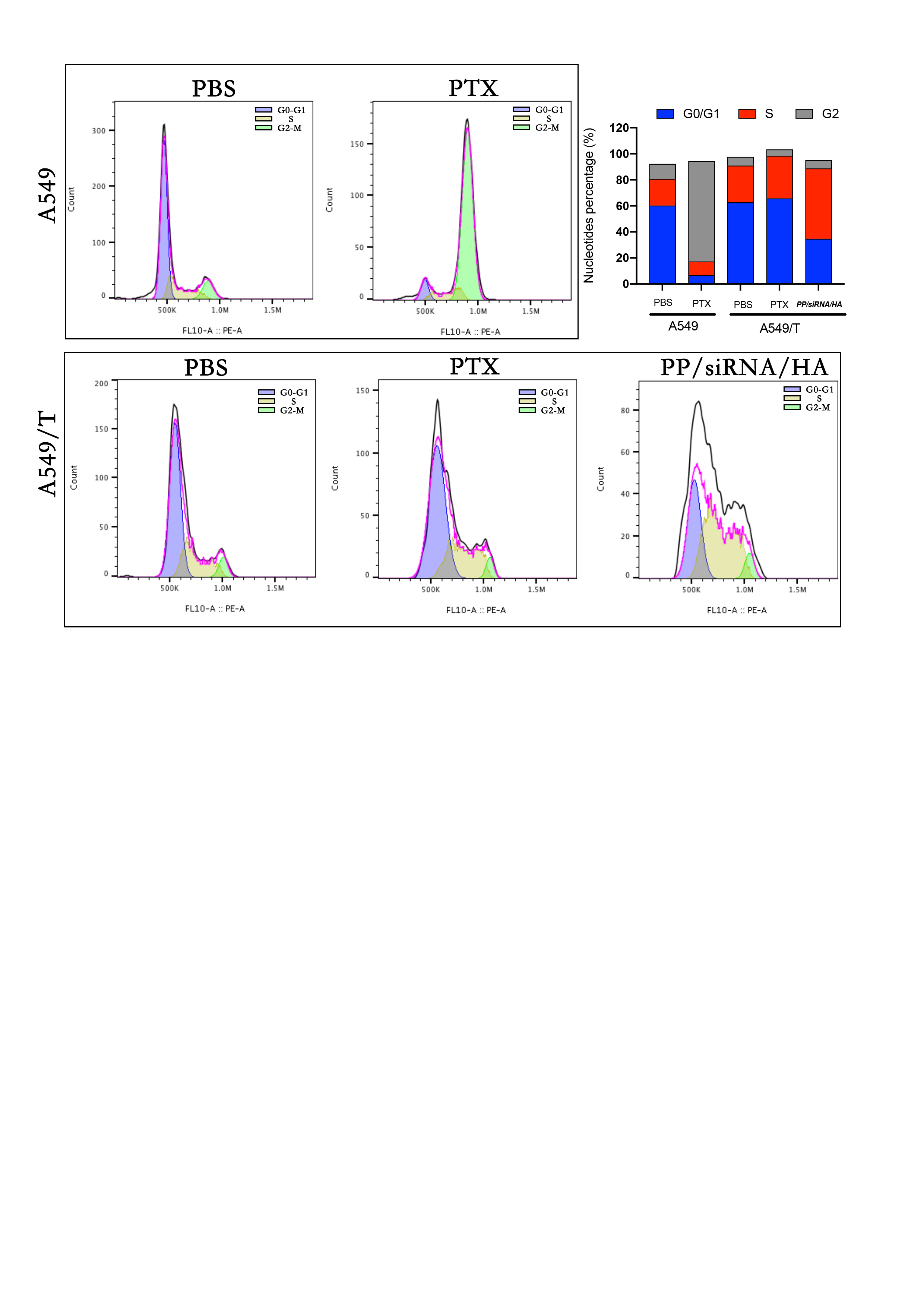


A

B

C

**Figure S18**. The cell-cycle distribution of A549 cells (A) and A549/T cells (B), and quantitive presentation of cell-cycle (C).


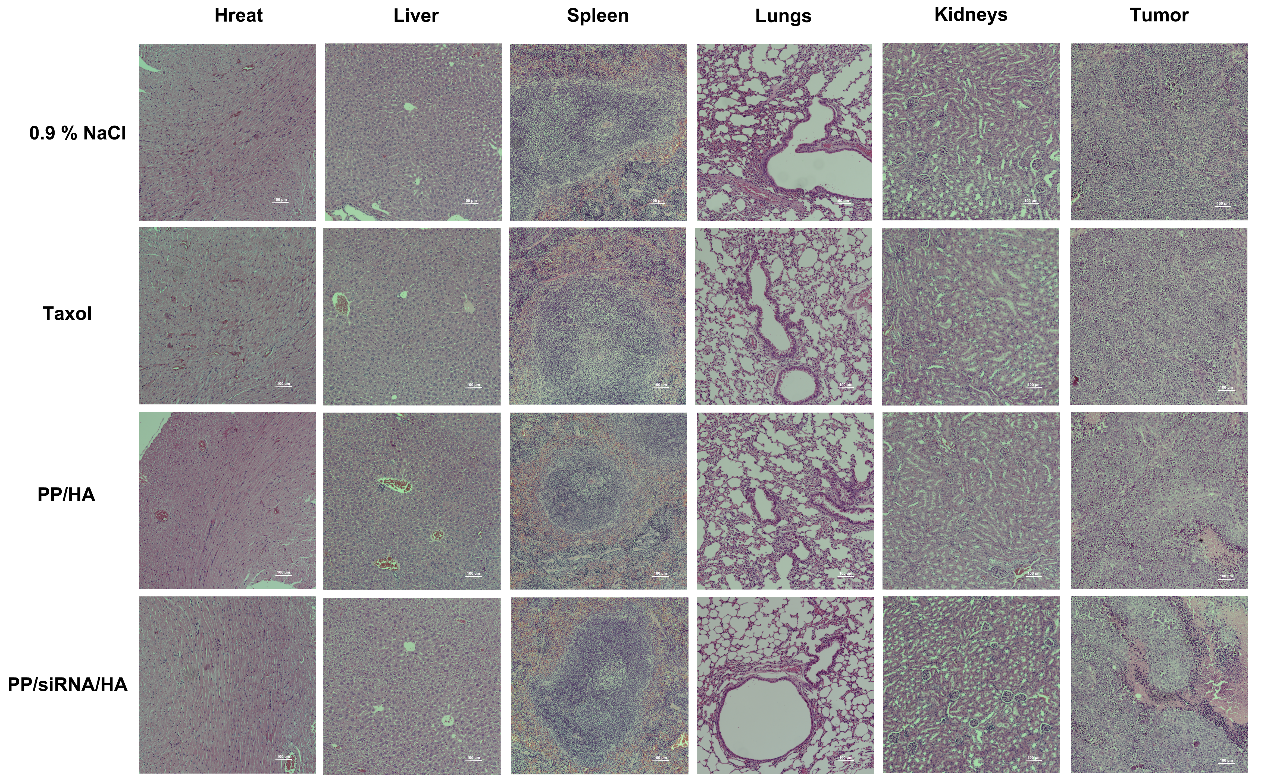
**Figure S19**. H&E staining of tumor tissues. Scale bars: 100 μm.
